# Prevention of functional and cognitive impairment through a multicomponent exercise program during and after hospitalization of older adults (PREDISC): Study protocol for a multicenter randomized clinical trial

**DOI:** 10.1371/journal.pone.0332391

**Published:** 2025-09-25

**Authors:** Nicolás Martínez-Velilla, Rosário Santos Silva, Patricia Alvarez Rodriguez, Jan Missé Xandri, Ana Pais, Encarna Ulloa Rodríguez, Gabor Abellan van Kan, Eva Peyrusque, Maite Izco-Cubero, Marisa Fernández González de la Riva, Regina Belo, Fabiola Zambom-Ferraresi, Iciar Echeverria Beistegui, Chenhui Chenhuichen, Arkaitz Galbete, Ana Isabel Gomes, Eva Heras, Fabricio Zambom-Ferraresi

**Affiliations:** 1 Department of Geriatrics, Navarre Health Service (SNS-O), Navarre University Hospital (HUN), Navarrabiomed, Navarre Public University (UPNA), Navarra Institute for Health Research (IdiSNA), Pamplona, Spain; 2 CIBER of Frailty and Healthy Aging (CIBERFES), Instituto de Salud Carlos III, Madrid, Spain; 3 Geriatrics and Active Aging Research Group (INGEA), Navarrabiomed, Pamplona, Navarra, Spain; 4 Unidade Local de Saúde do Baixo Mondego, Portugal; 5 Universidad Pública de Navarra, Pamplona, Navarra, Spain; 6 Servei Envelliment i Salut Servei Andorrà d’Atenció Sanitària, Servei Andorrà dÁtenció Sanitàriaorra la Vella, Andorra; 7 Gérontopôle of Toulouse, Institute on Aging, Toulouse University Hospital (CHU Toulouse), Toulouse, France; 8 IHU HealthAge, Toulouse University Hospital, La Cité de la Santé, Hôpital La Grave, Place Lange, Toulouse, France; PLOS: Public Library of Science, UNITED KINGDOM OF GREAT BRITAIN AND NORTHERN IRELAND

## Abstract

Hospitalisation in older adults is frequently associated with a significant risk of functional and cognitive decline. This study aims to evaluate the effectiveness of a multicomponent exercise program designed to prevent such declines in acutely hospitalised older adults. The intervention will be implemented across three European hospitals with the goal of enhancing the quality of life and functional independence of this vulnerable population. This study will be conducted as a multicentre, randomised controlled trial with two arms: one group will receive a multicomponent exercise program, while the control group will receive standard care. The intervention will consist of individualised sessions focusing on resistance training, balance, and aerobic exercises over four days during hospitalisation. The primary outcome will focus on functional capacity, which will be assessed using the Short Physical Performance Battery. The primary outcome will be measured at baseline, discharge, and 30- and 90-days post-discharge. In addition to this primary outcome, secondary outcomes will include the Barthel Index, cognitive function assessed through the Mini-Mental State Examination, quality of life measured by the EuroQol-5D-3L, and psychological well-being evaluated using the Geriatric Depression Scale (GDS). Additional metrics will include length of hospital stay and readmission rates within 30- and 90-days post-discharge.

**Trial registration:** ClinicalTrials.gov NCT06634147.

## Introduction

Functional decline among older adults during hospitalisation is a significant concern that affects a substantial portion of this demographic and correlates with adverse prognostic outcomes [1]. Recent research indicates that more than 50% of hospitalised older adults do not recover their pre-admission functional status within one year, leading to increased dependency, higher rates of institutionalisation, and elevated mortality rates [2–5]. This issue persists despite the evidence and recommendations established over 75 years ago [6].

Other than the clinical reason for admission, the observed decline is attributed to several factors, including extended immobilisation, insufficient promotion of physical activity, and disruption of the patient’s daily routine environment, which often emphasises the treatment of acute diseases over the preservation of the patient’s functional abilities. Physical inactivity during hospitalisation not only contributes to the loss of muscle mass, strength, and endurance but is also associated with cognitive decline and an increased incidence of secondary complications [2]. The hospital environment, often characterised by a medical approach favouring bed rest instead of promoting functional recuperation for older inpatients, exacerbates these issues, resulting in a cycle of disability that can lead to irreversible mobility disability.

In hospital settings, standard care practices encompass early mobilisation and physical therapy; however, these interventions frequently prove inadequate for preventing functional decline [7–10]. The limitations of current methodologies are apparent: a shortage of trained personnel to administer structured exercise programs, resource constraints, and a prevailing inclination to keep patients at rest because of concerns about falls. Furthermore, many non-specific rehabilitation programs are characterised by low intensity and are not customised to address the specific needs of older adults, thereby diminishing their efficacy [11].

Our team initially spearheaded a project at a tertiary hospital in Spain [12] which subsequently evolved into a multicentre study at the national level [13]. This project compellingly demonstrated the advantages of implementing an individualised multicomponent exercise program tailored to the specific characteristics and needs of each patient, even during the acute phase of the illness. The accumulated evidence indicates that a more comprehensive and innovative approach, which not only emphasises mobility but also incorporates resistance training and functional exercises adapted to each patient’s individual capabilities, is essential for preserving functional independence and enhancing the quality of life of hospitalised older adults [14–17].

Despite the increasing body of evidence supporting the advantages of exercise in older populations, a significant research gap persists regarding exercise interventions during acute hospitalisation. This study aims to assess the effectiveness of a multicomponent exercise program specifically tailored for hospitalised older adults, suggesting that the implementation of this program would not only enhance functional and cognitive capacities during hospitalisation but also facilitate and improve recovery after discharge, reducing falls, hospital readmission, and costs.

This study endeavours to address the existing gap in the literature regarding the efficacy of structured exercise programmes within the hospital context and their validation in an international setting. Through a rigorously designed intervention, we aim to demonstrate that a proactive approach through exercise can reverse the functional decline associated with hospitalisation, thereby promoting a shift in clinical practice towards a model that prioritises the functional health of older adults in acute care environments.

## Materials and methods

### Trial design

**Fig 1:**
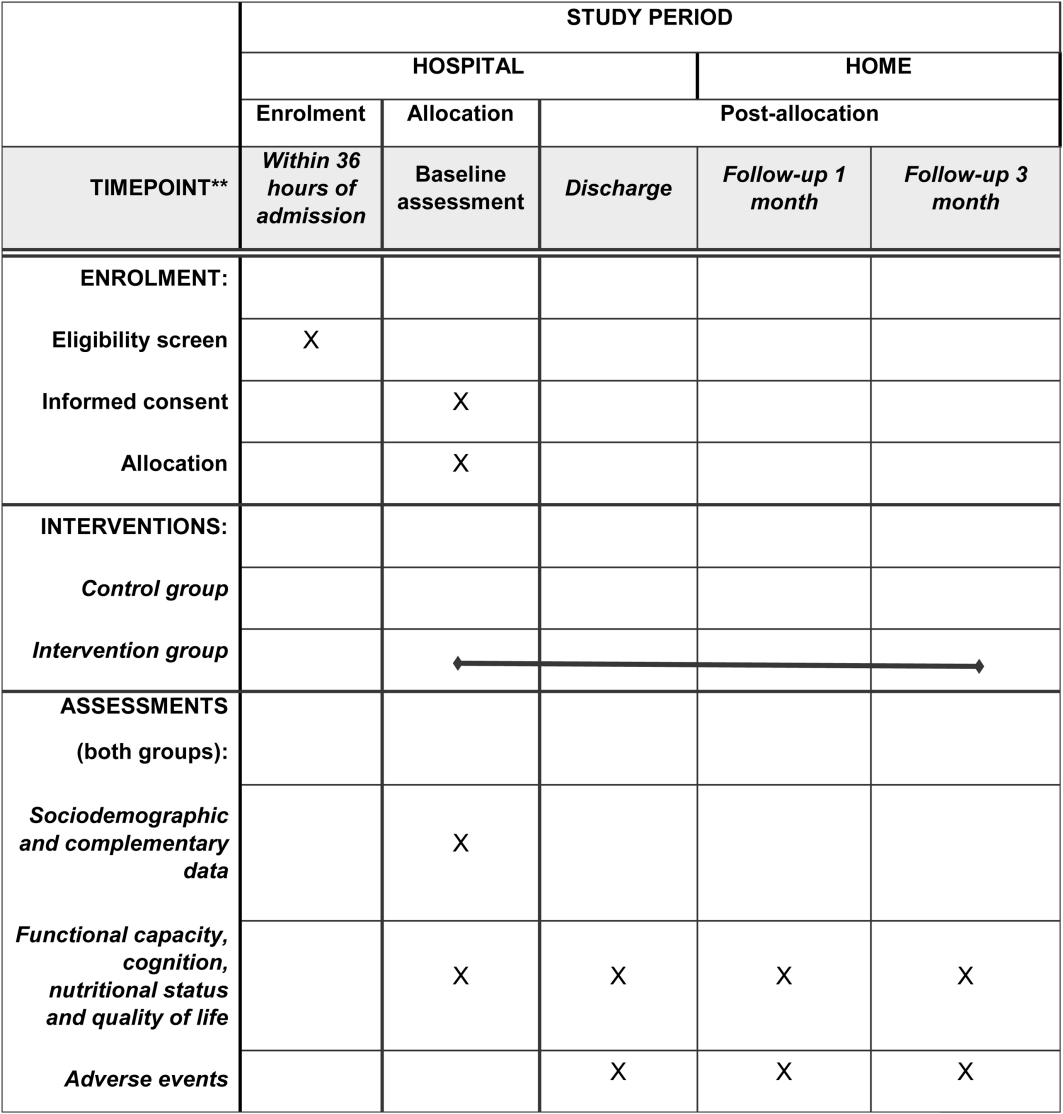
Outline of study as per SPIRIT checklist.

**Fig 2:**
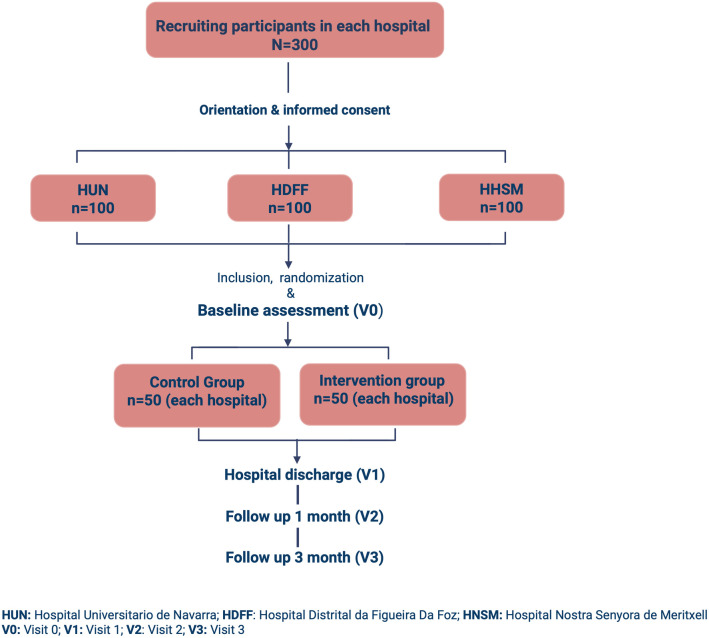
Flow chart of the study.

This study will be conducted in accordance with the Consolidated Standards of Reporting Trials [18]. This two-armed, multicentre randomised controlled trial (RCT) will evaluate the effectiveness of a multicomponent exercise program in preventing functional and cognitive decline among hospitalised older adults. The trial will be conducted at three European hospitals: Hospital Universitario de Navarra in Spain (HUN), Hospital Distrital da Figueira da Foz in Portugal (HDFF), and Hospital Nostra Senyora de Meritxell in Andorra (HNSM). A summary of enrolment, interventions and assessments is presented in Fig 1 (SPIRIT schedule), and a flowchart of the participants in Fig 2.

Eligible subjects will be invited to participate in the trial within 36 h of admission, and the baseline examination will be conducted directly after the inclusion evaluation of the clinical status and stability by the medical team. This trial was registered at ClinicalTrials.gov (NCT06634147). This protocol is reported in accordance with the Standard Protocols Items: Recommendations for Interventional Trials [19]. (S1 File, SPIRIT Checklist).

### Inclusion and exclusion criteria

Participants in this study will be older adults aged 75 years and above who are hospitalised in the Geriatrics and Internal Medicine departments for medical conditions. The inclusion criteria require participants to be able to ambulate with or without assistance, capable of providing informed consent or having a legal representative, and currently hospitalised due to a medical condition. Participation in the study is voluntary, and patients who do not wish to participate will not have their usual medical care affected. Conversely, individuals will be excluded if they have a life expectancy of less than three months, suffer from terminal illnesses, cannot participate in a multicomponent exercise program, exhibit moderate to severe cognitive impairment (Global Deterioration Scale, GDS, stages 6–7), or demonstrate moderate to severe disability (Barthel Index < 60). The eligibility criteria are presented in Table 1. Randomisation will be performed using an online random number generator to ensure the unbiased allocation of participants to the intervention and control groups (randomizer.org).

**Table 1:** Eligibility criteria.

INCLUSION CRITERIA	• ≥ 75 years old hospitalized in European Geriatrics and Internal Medicine services (HUN, HDFF and HNSM) • Expected hospital stay > 6 days
EXCLUSION CRITERIA	• Refusal to sign the informed consent by the patient/primary caregiver/legal guardian or inability to obtain it. • Life expectancy of less than 3 months or oncologic or non-oncologic disease in terminal phase. • Impossibility of follow-up. • Inability to participate in a multicomponent exercise program. • Medical contraindication to exercise. • Moderate-severe stage neurocognitive disorder GDS – Fast Reisberg 6–7 • Moderate-severe disability (measured by Barthel Index (BI < 60).

### Intervention

Patients in the control group will receive standard care currently provided to all patients, including referrals to physiotherapy as needed. The recruitment start date will be January 2025 in Spain, and September 2025 in Andorra and Portugal, and the expected end date is June 2026. In contrast, the intervention group will participate in a structured multicomponent exercise program, which will encompass progressive and supervised training combining aerobic resistance, strength, and balance over a duration of 4 days during their hospitalisation. The multicomponent training protocol is presented in Table 2. This program will be overseen by trained and experienced professionals on the research team, and upon discharge, participants will receive individualised guidelines for continuing the multicomponent exercise program for three months, along with personalised nutritional recommendations.

**Table 2:** Multicomponent training protocol.

WARM UP								
Stationary bicycle*						Chair squats*		
5 min (Borg 3–4)						3 sets x 10 rep.		
RESISTANCE TRAINING*								
Exercises								
Leg press – Seated bench press machine – Leg extension								
PROGRESSION WEIGHT PROTOCOL								
Assessment	**V0**					**V1**	**V2**	**V3**
Session	1RM	S1*	S2*	S3*	S4*	1RM	1RM	1RM
Sets		2	3	3	3			
Repetition		10	10	10	8			
(% 1RM)		40	50	60	70			
Rest (sec)		120 between sets and exercises						
BALANCE: Core and Hip stability*								

*Only Intervention Group.

**V0: visit 0; V1: visit 1; V2: visit 2; V3: visit 3; S1: session 1; S2: session 2; S3: session 3; S4: session 4.**

The multicomponent exercise program will include chair squats (sit-to-stand exercise). The main training phase will use machines for strength training targeting major muscle groups: the lower extremities (such as leg press and knee extension) and one exercise for the upper extremities (seated chest press). The objective is to perform 2–3 sets of 8–10 repetitions at an intensity of 40–70% of one repetition maximum (1RM), tailored to everyone’s functional capacity. Each training session will also incorporate hip abduction exercises, balance exercises, and stretching routines, ensuring a comprehensive approach to enhance health and mobility among participants. A daily logbook will be used during the hospital stay to register the exercise sessions performed for the intervention group, as well as treatment safety.

**Fig 3:**
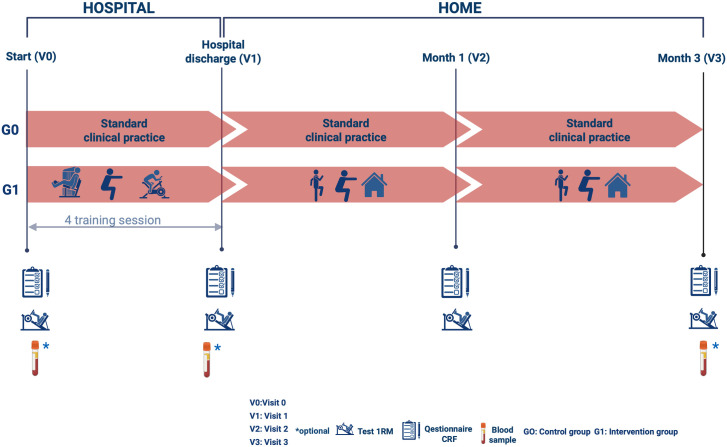
Schematic study timeline.

Patients will be followed up for 3 months, and there will be coordination between the exercise program in the hospital and primary care (Fig 3).

### Data collection

Data will be systematically collected at four distinct time points to evaluate the intervention effects. Initially, at admission, a comprehensive assessment will be conducted to establish baseline measures of functional capacity, cognition, nutritional status, and quality of life. Following this, at discharge, the same variables will be re-evaluated to determine any immediate changes resulting from the intervention. Additionally, follow-up assessments will be performed at one- and three-months post-discharge to monitor longitudinal changes in functional capacity, cognition, quality of life, and to document any adverse events, including falls and readmissions. This structured approach aims to provide a robust dataset for analysing the efficacy of the intervention over time.

The **primary outcome** of the study focuses on the functional capacity of older adults, assessed through the Short Physical Performance Battery (SPPB), with evaluations occurring at discharge, one month, and three months post-discharge compared to baseline. The SPPB consists of three essential components: standing balance, walking speed, and the 5 times chair stand test, which assesses lower extremity strength and endurance. This tool has been rigorously validated in older adults experiencing frailty, demonstrating high sensitivity to changes in their physical function. Furthermore, the SPPB is a reliable predictor of various clinical outcomes, making it a valuable instrument for evaluating the functional capabilities of older adults [20]. **Secondary outcomes** encompass a variety of health-related variables, including changes in Barthel Index [21] scores and the percentage of functional recovery after hospitalization. Additionally, cognitive variables such as the Mini-Mental State Examination (MMSE) [22] and the Trail Making Test A (TMT-A) [23] will be assessed from baseline to discharge, and at one and three months. Changes in affective status (GDS), [24] number of medications, fall frequency, mortality, hospital readmissions, quality of life (EuroQoL-5D-3L) [25], maximum isometric strength (HandGrip), and maximum dynamic strength in the upper and lower limbs will also be assessed, along with the average length of hospital stay and changes in sleep quality (SATED scale) [26] at discharge, at one month, and at three months. The assessment times for the different variables are shown in Table 3.

**Table 3:** Time of assessment of different variables in the trial.

MEASUREMENT	DURING HOSPITALIZATION	AFTER DISCHARGE		
	Visit 0	Visit 1	Visit 2	Visit 3
	(inclusion)	(discharge)	(1 month)	(3 month)
**Sociodemographic information**	**x**			
**Height and weight**	**x**		**x**	**x**
**Adverse events**		**x**	**x**	**x**
**Previous lifestyles questions (Alcohol, tobacco..)**	**x**			
**Mini nutritional assessment (MNA-30)**	**x**			
**SARC-F**	**x**			
**Barthel Index**	**x**	**x**	**x**	**x**
**Short Physical performance Battery (SPPB)**	**x**	**x**	**x**	**x**
**Test 1RM**	**X**	**X**	**X**	**X**
**HandGrip**	**x**	**x**	**x**	**x**
**Linda fried frailty**	**x**			
**Mini-Mental State Examination (MMSE)**	**x**	**x**	**x**	**x**
**Trail Making Test Part a (TMT-A)**	**x**	**x**	**x**	**x**
**Yesavage Geriatric Depression Scale (GDS-15)**	**x**	**x**	**X**	**X**
**Delirium 4AT**	**x**	**x**		
**Reisberg Scale (GDS)**	**x**			**x**
**Quality of life (EQ-5D-3L)**	**x**		**x**	**x**
**SATED Scale**	**x**	**x**	**x**	**x**
**Cumulative Illness Rating for Geriatrics (CIRS-G)**	**x**			
**Polypharmacy**	**x**			
**Falls**	**x**	**x**	**x**	**x**
**Personal history and other geriatric syndromes. Diseases considered grouped by CIE-10 codes and by Salisbury ACG codes.**	**x**			

Peripheral blood (PB) samples will be obtained from all patients at baseline (V0), at discharge (V1), and at 90 days (V3) to obtain serum, plasma, and buffy coat after centrifugation in a fixed-angle rotor at 3,300 *rpm* for 10 min at room temperature. Samples will be divided into 100 μL aliquots and immediately stored at −80°C.

### Data analysis

#### Power of the trial: Sample size and power calculation.

We calculated the required sample size to detect a statistically significant difference in the Short Physical Performance Battery (SPPB) scores following the intervention. A significance level (α) of 5% was assumed, with a correlation coefficient (ρ) of 0.5 between the pre- and post-intervention values, and a standard deviation (σ) of 2.5 for the SPPB. The required sample size to achieve a statistical power of 90% and detect a clinically significant minimum difference of one point in the post-intervention SPPB score was determined to be 102 patients per group. To account for an anticipated dropout rate of 30% during follow-up, the final required sample size increased to 148 patients per group, resulting in a total of 296 participants across both groups. This calculation implies the need to recruit approximately 50 patients per group at each of the three participating hospitals (N = 300).

### Statistical methods

First, a comprehensive descriptive analysis will be conducted on the entire sample and within individual groups. For continuous variables, measures of central tendency and dispersion will be calculated based on their distribution, utilising either the mean and standard deviation or the median and interquartile range (IQR). Qualitative variables will be summarised as frequencies and percentages, with corresponding confidence intervals to estimate the overall prevalence. Baseline characteristics between the treatment and control groups will be compared using the Student’s t-test or the Mann–Whitney U test for quantitative variables, and the Chi-square test or Fisher’s exact test for qualitative variables.

The impact of the intervention on various outcome measures will be assessed using ANCOVA or linear mixed-effects models with a random intercept for continuous variables. Logistic regression models and analyses of rate or proportion differences will be applied to outcomes such as falls, mortality, and institutionalisation. In the event of significant baseline imbalances in any independent variable, sensitivity analyses will be conducted to adjust for these differences in the final analysis. No imputation of missing data will be conducted.

The analysis will adhere to the intention-to-treat principle, and additional per-protocol analyses will be performed as a secondary sensitivity measure. Statistical significance will be determined at a threshold of 0.05 for all tests. Data analysis will be conducted using SPSS (version 25.0) and R software (version 4.0).

### Patient and public involvement

The trial is designed through a collaborative dialogue involving researchers, clinicians, and healthcare managers from various disciplines. Although physical activity professionals will primarily lead the interventions, effective geriatric care requires a multidisciplinary approach to address the complex health needs of older adults, who often present with multiple concurrent health issues. This interdisciplinary and practice-oriented framework enhances the likelihood of successful intervention implementation, should they prove effective. Qualitative interviews will also be conducted with participants and their families from both the intervention and control groups to gain insights into their experiences. These interviews will take place either in the hospital during their stay or at the participants’ homes following discharge, ensuring a comprehensive understanding of their perspectives and the impact of the interventions on their health.

### Ethics and dissemination

Ethical approval for this trial has been granted by the Clinical Research Ethics Committees: “Comité de Ética De Ética De La Investigación Con Medicamentos de Navarra” (PI_2024/47; may 10, 2024; November 7, 2024), “Centro de Investigação Clínica CiC2Fs” (26.OBS.2024) and Comissió Ètica de la Investigació (02-2025-CEI-PREDISC, April 24, 2025), Data collection will be conducted with careful consideration of information requirements, informed consent, confidentiality obligations, and data utilization requirements. Participants will receive comprehensive verbal and written information about the study, and informed written consent will be obtained by the research staff prior to participation. All collected data will be pseudonymized and stored in both paper and digital formats, in accordance with the General Data Protection Regulation. Only the responsible researchers will have full access to the data. Trial findings will be disseminated through multiple channels, including scientific publications, conferences, and workshops, aimed at healthcare professionals and the public. We will adhere to the Vancouver recommendations for the authorship.

### Trial status

Participant recruitment is expected to be completed by January 2026. Data collection is scheduled to conclude in March 2026, and the study results are anticipated to be available by June 2026. Any deviations from this protocol will be updated on ClinicalTrials.gov, and changes will be reported when disseminating the results.

## Discussion

The primary objective of this trial is to advance the understanding of strategies aimed at preventing physical decline in acutely hospitalised patients aged ≥ 75 years. Although research has demonstrated the benefits of community-based exercise programs for older adults [26,27], there is a paucity of studies focusing on exercise interventions during hospitalisation for acute medical conditions. Currently, there is no consensus on the most effective exercise regimen for mitigating functional deterioration in this population [11]. Older adults represent a significant portion of healthcare expenditures; however, clinical guidelines often rely on studies involving younger individuals without severe comorbidities. This lack of age-appropriate research underscores the need for tailored interventions for older patients, particularly those experiencing frailty.

Our trial aims to introduce an approach for preserving physical function in older adults. If the findings indicate positive outcomes, we will explore the implementation and cost-effectiveness in future studies. A successful intervention could revolutionise clinical practice by integrating multi-component exercise routines into standard care. Emphasising physical function is crucial, as it serves as an indicator of healthy aging and predicts future health status and mortality after hospitalisation. Our interventions were informed by two prior studies conducted with hospitalised older adults in Spain [12,13]. These studies have demonstrated that multicomponent exercise programs are safe and effective in reducing functional limitations in older adults. However, further research is necessary to assess the applicability of these findings in diverse healthcare settings and countries. In this trial, we increased the daily exercise duration from 20 minutes to 20–30 minutes, aligning with Ortiz-Alonso et al.‘s findings to ensure comparability in daily exercise time during hospitalisation [17]. The interventions will be conducted on all weekdays, when possible, to ensure the maximal benefit of the intervention.

We anticipate that this trial will identify the most effective exercise program for implementation in the future. Each exercise regimen has unique advantages and disadvantages. While a comprehensive program may yield better immediate results in physical function at discharge owing to its individualised nature, it may be less conducive to self-training than simpler programs. A simpler program, more amenable to self-directed training, might demonstrate equal or superior effectiveness in maintaining physical function three months post-discharge, as participants can continue independently at home. Future analyses will explore the correlations between baseline characteristics and the effectiveness of the interventions.

Future studies should investigate the feasibility of transitional care interventions that integrate in-hospital physical exercise with home-based self-training supported by primary care services and assess their scalability across healthcare systems. This multicentre approach represents a critical step in identifying optimal strategies to sustain or enhance physical and cognitive functions in acutely hospitalised older adults.

## Conclusion

In conclusion, the PREDISC trial has the potential to provide valuable insights into methods for maintaining and improving physical function in acutely hospitalised older adults. As the aging population continues to grow, the number of hospital admissions among older adults is expected to increase. Therefore, identifying effective interventions to mitigate the negative health effects of acute hospitalisation is of utmost importance from both clinical and public health perspectives.

## Supporting information

S1 File Reporting checklist for protocol of a clinical trial (SPIRIT checklist). (DOCX)

S2 Doc Protocol approved by the Ethics Committe (english). (DOCX)

S3 Doc Protocol approved by the Ethics Committe (spanish). (DOCX)
