# Supplementary material for: Prevention of functional and cognitive impairment through a multicomponent exercise program during and after hospitalization of older adults (PREDISC): Study protocol for a multicenter randomized clinical trial
Source: PLoS One. 2025 Sep 25;20(9):e0332391. doi: 10.1371/journal.pone.0332391 (PMC12463233; doi:10.1371/journal.pone.0332391)
Supplement: S2 Doc — (DOCX) [file pone.0332391.s002.docx]

V1 – 10/05/2024



**PREDISC**

**Prevention of functional and cognitive decline through a multicomponent exercise program in hospitalized elderly patients (Geriatrics and Internal Medicine): randomized clinical trial. Multicenter study (HUN, CHU-T, ULSBM, and HNSM)**

**Geriatrics Department, HUN**

**INGEA – Research in Geriatrics and Active Aging – Navarrabiomed**

**Public University of Navarra**

**RESEARCHERS:**

UNIVERSITY HOSPITAL OF NAVARRA (HUN) - NAVARRABIOMED - UPNA, Navarra

Nicolás Martínez Velilla

Geriatrics Department

University Hospital of Navarra

Navarrabiomed – UPNA – IdiSNA

Fabricio Zambom Ferraresi

Navarrabiomed – UPNA – IdiSNA

Iciar Echeverría Beistegui

Navarrabiomed

Fabiola Zambom Ferraresi

Navarrabiomed

Marisa Fernández Gonzáles de la Riva

Navarrabiomed

Maite Izco Cubero

Navarrabiomed

**COLLABORATORS:**

CENTRE HOSPITALIER UNIVERSITAIRE DE TOULOUSE, GERIATRICS DEPARTMENT (CHU-T), Occitanie

Gabor Abellan Eva Peyrusque

Cécile Dedieu

UNIDADE LOCAL DE SAUDE BAIXO MONDEGO (ULSBM), Coimbra

A. Isabel Gomes Ana Isabel Pais

Maria do Rosário Pereira Costa Santos Silva

HOSPITAL NOSTRA SENYORA DE MERITXELL (HNSM), Aging and Health Services of SAAS, Andorra

Eva Heras Jan Missé

Encarna Ulloa Maria Anglada

**RESEARCH PROJECT SUMMARY**

| PROMOTOR | Navarrabiomed. Hospital Universitario de Navarra. Universidad Pública de Navarra |
| --- | --- |
| DIRECTOR Y SUPERVISOR  DEL ESTUDIO DE INVESTIGACIÓN | Nicolás Martínez Velilla |
| TITLE | PREDISC: Prevention of functional and cognitive decline through a multicomponent exercise program in hospitalized elderly patients (Geriatrics and Internal Medicine): randomized clinical trial. Multicenter study (HUN, CHU-T, ULSBM, and HNSM). |
| OBJECTIVES | General objective: To analyze whether a multicomponent training program intervention in people aged 75 years or older hospitalized for a medical condition improves functional capacity.  Specific objectives:  1. To analyze the patient subgroups that benefit most from the intervention (frail, disabled, cognitively impaired, malnourished patients, etc.), identifying patient factors (sociodemographic, clinical, functional, and cognitive) and intervention factors (exercise type, intensity, frequency) that could explain differences in the intervention's effectiveness.  2. To analyze whether a multicomponent training program intervention in people aged 75 years or older hospitalized for a medical condition improves cognitive status during follow-up and after hospital discharge.  3. To analyze whether a multicomponent exercise program intervention in people aged 75 years or older hospitalized for a medical condition reduces drug use and iatrogenic drug use.  4. To monitor the changes achieved through the intervention, as well as the factors that determine the continuation of the benefits of exercise in the short and medium term.  5. To analyze the costs (comparing average length of stay, readmissions, institutionalization, and use of healthcare resources) of the population that exercises compared to the population that does not.  6. To examine the effect of a strength, balance, and gait training program in hospitalized patients on muscle mass, balance ability, muscle power, and gait speed in a clinical setting.  7. To analyze whether an intervention using a multicomponent training program in people aged 75 years or older hospitalized for a medical condition improves participants' quality of life.  8. To analyze whether an intervention using a multicomponent training program in people aged 75 years or older hospitalized for a medical condition improves participants' sleep quality.  9. Analyze whether an intervention using a multicomponent training program in people aged 75 years or older hospitalized due to a medical illness produces changes at the Omics level. |
| DESIGN | Multicenter Randomized Clinical Trial |
| INCLUSION CRITERIA | - People aged 75 years or older hospitalized in European Geriatrics and Internal Medicine services (HUN, CHU-T, ULSBM, and HNSM).  - Able to communicate.  - Able to ambulate, with or without personal/technical assistance, or able to move unaided in a wheelchair. |
| EXCLUSION CRITERIA | - Refusal to sign informed consent by the patient/primary caregiver/legal guardian or inability to obtain it.  - Life expectancy of less than 3 months or terminally ill oncological or non-oncological disease.  - Inability to be followed up.  - Inability to participate in a multicomponent exercise program.  - Medical contraindication to exercise.  - Moderate to severe neurocognitive disorder (GDS - Fast Reisberg 5-7).  - Moderate to severe disability (measured by the Barthel Index [BI] <60).  - Expected hospitalization of less than 6 days. |
| SIZE | 296 patients across the 4 participating centers – 74 patients in Navarra (37 control group and 37 intervention group) |
| DURATION OF THE STUDY | 30 months |

**INTRODUCCIÓN**

The progressive aging of society and the challenge of caring for the elderly have become a truly urgent matter for all countries and healthcare systems. The elderly are increasingly taking center stage in the activities of healthcare and social services, and their needs are completely different from those of the adult population and those of 50 years ago. In this regard, one of the world's leading health organizations, the World Health Organization (WHO), recently published the first "World Report on Ageing and Health." One of the report's major new features is the introduction of the concept of intrinsic capacity, understood as the set of physical and mental capacities that equip individuals with functional skills that enable them to face the challenges posed by the environment. One of the report's key messages is that health systems serving the elderly should shift their focus from disease to intrinsic capacity (i.e., function). This is particularly important in the care of vulnerable older adults at risk of disability (those who are frail and have cognitive impairment), in order to be able to respond early.

Frailty can be defined as a clinical condition in which there is an increased risk of individual vulnerability to developing adverse events such as dependency and/or mortality when exposed to stressors. A recent international consensus defines physical frailty as a major "medical syndrome with multiple causes and contributors characterized by decreased strength, endurance, and physiological function that increases individual vulnerability to developing further dependency and/or death." The prevalence of this syndrome is high in individuals over 65 years of age, depending on the screening method used (7–16%), and increases with age. The most established consensus on the diagnostic phenotype encompasses several domains, including deterioration in physical function (slowed gait speed, fatigue, decreased grip strength), weight loss, and low physical activity. Linked to the aging phenomenon, the functional capacity of the neuromuscular, cardiovascular, and respiratory systems begins to progressively decline, leading to an increased risk of frailty.

The traditional approach to the concept of frailty has been based on a clinical phenotype, but biochemical and molecular knowledge has gradually been incorporated. For example, it is important to highlight the relationship between low IGF-1 levels and sarcopenia and disability, as well as the inverse relationship between inflammatory factors such as interleukin 6 and its relationship with muscle proteolysis, disability, and mortality. On the other hand, despite growing evidence of the role of the microbiota in organisms, no link has yet been established with the vulnerability of the elderly. The integrity of the intestinal epithelium protects the body from aggression. Injury to or loss of the intestinal barrier can trigger the development of different types of inflammatory bowel diseases and some autoimmune diseases. To date, there is limited information regarding microbiota modification in hospitalized settings, as well as the protective effects that a multicomponent exercise program may have. Hospitalized elderly patients are affected by a series of circumstances unrelated to the pathological process that led to their hospitalization, which typically worsen hospitalization outcomes. In fact, the implementation of care models other than traditional ones has shown clear benefits in the short- and medium-term functional outcomes of these patients. However, the components of these models that explain these improved outcomes have not been evaluated differentially. Some examples of these circumstances include the routine orders of absolute bed rest without any objective criteria for patients who are able to ambulate, the perpetuation of continuous intravenous fluid therapy, physical and chemical restrictions, unnecessary indwelling catheters, etc. On numerous occasions, hospitalized elderly patients spend most of their time in bed, with the rate even higher than 83% of those confined to bed compared to 4% of those who are able to stand or walk. This population, with reduced functional and physiological reserve, is more vulnerable to the effects of bed rest, for example, which range from functional loss or cognitive decline to longer stays, mortality and institutionalization, worse mood, delirium, deconditioning, aspirations, pressure ulcers and falls, decreased caloric intake, social isolation, poorer quality of life, and greater use of health-related resources.

The benefits of physical exercise in aging, and specifically in frailty, have been the subject of recent scientific research. Thus, it has been proven that increased physical activity in the elderly has been associated with a decreased risk of mortality, the risk of chronic diseases prevalent in aging (cardiovascular, osteoarticular, neurodegenerative), institutionalization, and functional decline. More specifically, the most beneficial type of physical exercise for frail elderly patients is the so-called multicomponent type. This type of program combines strength, endurance, balance, and gait training and is the one that has demonstrated the most significant improvements in functional capacity, which is a fundamental element for maintaining independence in basic and instrumental activities of daily living in older adults.

Given the aforementioned relationship between frailty and cognitive decline, it makes sense that interventions that are effective in frail patients could be beneficial in older adults with cognitive decline, and vice versa. Although studies are scarce, recent studies demonstrate how weekly resistance exercise programs for 12 weeks in a cohort of elderly women not only lead to increases in walking speed but also benefit from improving executive cognitive functions, which are interestingly related to the risk of falls.

In our research group, we have corroborated these results by implementing multicomponent exercise programs in institutionalized frail elderly patients with mild cognitive impairment or dementia, with significant improvements in functional capacity, risk of falls, and executive cognitive functions. Other authors have found similar results in terms of reversal of frailty and cognitive improvements, although patients with initial cognitive impairment are often excluded at baseline, making it difficult to extrapolate the results to a significant percentage of elderly patients.

However, despite the overwhelming scientific evidence that physical activity improves the health of the population, society in general, and healthcare professionals in particular, it is still not fully implemented in our healthcare systems. This would necessarily imply an individualized prescription according to the functional capacity of the elderly person with specific recommendations on dosage (intensity, volume, frequency) as is done with other medications, with the advantage that the iatrogenesis underlying the drugs does not exist with exercise.

We consider the European VIVIFRAIL exercise prescription program (www.vivifrail.com), which will be implemented in this study and which was developed by world-class experts in the field of physical activity and frailty (who are part of the research team, including the program coordinator), to be a milestone in this regard. For the first time, significant progress has been made in the aforementioned individualized "exercise prescription" and its application to a population as vulnerable as frail elderly patients with cognitive impairments. We believe this could represent a significant advance in improving the functional capacity of these patients and, as the WHO states, should be the objective of healthcare systems that care for these individuals.

If our hypothesis is correct, it opens the door to modifying the current hospitalization and outpatient management system for elderly patients. If we modify current guidelines, it is likely that older adults will experience lower levels of functional and cognitive decline after admission, and predictably a better quality of life and lower healthcare resource consumption (fewer readmissions, less institutionalization, among others). Furthermore, while exercise interventions in older patients have typically been delivered to participants in the community, institutions, or hospitalized for rehabilitation, there is a lack of evidence for older patients with acute medical conditions, who often have multiple comorbidities.

A very important aspect of our study is that the only exclusion criteria (unlike most previous studies) allow for the participation of older adults with different cognitive and functional levels, as the sole premise is that they can ambulate and actively participate in the program.

References:

- Martínez-Velilla N, Casas-Herrero A, Zambom-Ferraresi F, Sáez de Asteasu ML, Lucia A, Galbete A, García-Baztán A, Alonso-Renedo J, González-Glaría B, Gonzalo-Lázaro M, Apezteguía Iráizoz I, Gutiérrez-Valencia M, Rodríguez-Mañas L, Izquierdo M. Effect of Exercise Intervention on Functional Decline in Very Elderly Patients During Acute Hospitalization: A Randomized Clinical Trial. JAMA Intern Med (IF: 21.87; Q1). 2019 Jan 1;179(1):28-36. doi: 10.1001/jamainternmed.2018.4869. PMID: 30419096
- Martínez-Velilla N, Abizanda P, Gómez-Pavón J, Zambom-Ferraresi F, Sáez de Asteasu ML, Fiatarone Singh M, Izquierdo M. Effect of an Exercise Intervention on Functional Decline in Very Old Patients During Acute Hospitalizations: Results of a Multicenter, Randomized Clinical Trial. JAMA Intern Med (IF: 21.87; Q1). 2022 Mar 1;182(3):345-347. doi: 10.1001/jamainternmed.2021.7654. PMID: 35040873
- Word Health Organization (2015). World Report on Aging on Ageing. Geneva. ISBN: 978 9241565042
- Clegg A, Joung G, Lille S et al. Frailty in elderly people. Lancet 2013: 381: 752-762
- Rodriguez Mañas L., Fried L. Frailty in the clinical scenario. Lancet 2015; 385: e7-e9.
- Robertson DA, Savva GM, Coen RF, Kenny RA. Cognitive function in the prefrailty and frailty syndrome. J Am Geriatr Soc. 2014 Nov;62(11):2118-24.
- Cognitive frailty: rational and definition from an (I.A.N.A./I.A.G.G.) international consensus group.
- Kelaiditi E, Cesari M, Canevelli M, van Kan GA, Ousset PJ, Gillette-Guyonnet S et al ; IANA/IAGG.J Nutr Health Aging. 2013 Sep;17(9):726-34
- Liu-AmBrose t, NagaMatsuls, GraF P, Beattie Bl, asHe MC, HanDytC. Resistance training and executive functions: a 12-month randomized controlled trial. ArchInternMed 2010 25; 170: 170-178.
- Zheng G1, Xia R2, Zhou W2, Tao J2, Chen L3. Aerobic exercise ameliorates cognitive function in older adults with mild cognitive impairment: a systematic review and meta-analysis of randomised controlled trials. Br J SportsMed. 2016 Apr 19.
- Cadore EL, Moneo AB, Mensat MM, Muñoz AR, Casas-Herrero A, Rodriguez-Mañas L, Izquierdo M. Positive effects of resistance training in frail elderly patients with dementia after long-term physical restraint. Age (Dordr). 2014 Apr;36(2):801-11.
- Casas-Herrero A, Cadore EL, Zambom-Ferraresi F, Idoate F, Millor N, Martínez-Ramirez A, Gómez M, Rodriguez-Mañas L, Marcellán T, de GordoaAR,Marques MC, Izquierdo M. Functional capacity, muscle fat infiltration, power output, and cognitive impairment in institutionalized frail oldes told. Rejuvenation Res. 2013 Oct;16(5):396-403.
- Tarazona-Santabalbina FJ, Gómez-Cabrera MC, Pérez-Ros P, Martínez-Arnau FM, Cabo H,Tsaparaset al A Multicomponent Exercise Intervention that Reverses Frailty and Improves Cognition, Emotion, and Social Networking in the Community-Dwelling Frail Elderly: A Randomized Clinical Trial. Am MedDirAssoc. 2016 May 1;17(5):426-33.
- Volpato S, Onder G, Cavalieri M, et al. Characteristics of nondisabled older patients developing new disability associated with medical illnesses and hospitalization. J Gen Intern Med 2007, 22:668-674.
- Baztan JJ, Suarez-Garcia FM, Lopez-Arrieta J, et al. Effectiveness of geriatric units on functional decline, living at home, and case fatality among older patients admitted to hospital for acute medical disorders: meta-analysis. BMJ 2009, 338: b50.
- Covinsky KE, Palmer RM, Fortinsky RH, et al. Loss of independence in activities of daily living in older adults hospitalized with medical illnesses: increased vulnerability with age. J Am Geriatr Soc 2003, 51:451-458.
- Brown CJ, Redden DT, Flood KL, et al. The underrecognized epidemic of low mobility during hospitalization of older adults. J Am Geriatr Soc 2009, 57:1660-1665.
- Brown CJ, Friedkin RJ, Inouye SK. Prevalence and outcomes of low mobility in hospitalized older patients. J Am Geriatr Soc 2004, 52:1263-1270.
- Creditor MC. Hazards of hospitalization of the elderly. Ann Intern Med 1993, 118:219-223.
- Allman RM, Goode PS, Patrick MM, et al. Pressure ulcer risk factors among hospitalized patients with activity limitation. JAMA 1995, 273:865-870.
- Gillick MR, Serrell NA, Gillick LS. Adverse consequences of hospitalization in the elderly. Soc Sci Med 1982, 16:1033-1038.
- Fortinsky RH, Covinsky KE, Palmer RM, et al. Effects of functional status changes before and during hospitalization on nursing home admission of older adults. J Gerontol A Biol Sci Med Sci 1999, 54:M521-526.
- Narain P, Rubenstein LZ, Wieland GD, et al. Predictors of immediate and 6-month outcomes in hospitalized elderly patients. The importance of functional status. J Am Geriatr Soc 1988, 36:775-783.
- Hirsch CH, Sommers L, Olsen A, et al. The natural history of functional morbidity in hospitalized older patients. J Am Geriatr Soc 1990, 38:1296-1303.
- Inouye SK, Wagner DR, Acampora D, et al. A predictive index for functional decline in hospitalized elderly medical patients. J Gen Intern Med 1993, 8:645-652.
- Sager MA, Franke T, Inouye SK, et al. Functional outcomes of acute medical illness and hospitalization in older persons. Arch Intern Med 1996, 156:645-652.
- Sager MA, Rudberg MA, Jalaluddin M, et al. Hospital admission risk profile (HARP): identifying older patients at risk for functional decline following acute medical illness and hospitalization. J Am Geriatr Soc 1996, 44:251-257.
- Rudberg MA, Sager MA, Zhang J. Risk factors for nursing home use after hospitalization for medical illness. J Gerontol A Biol Sci Med Sci 1996, 51:M189-194.
- Covinsky KE, Justice AC, Rosenthal GE, et al. Measuring prognosis and case mix in hospitalized elders. The importance of functional status. J Gen Intern Med 1997, 12:203-208.
- Lakhan P, Jones M, Wilson A, et al. A prospective cohort study of geriatric syndromes among older medical patients admitted to acute care hospitals. J Am Geriatr Soc 2011, 59:2001-2008.
- Hannan EL, Magaziner J, Wang JJ, et al. Mortality and locomotion 6 months after hospitalization for hip fracture: risk factors and risk-adjusted hospital outcomes. JAMA 2001, 285:2736-2742.
- Kortebein P, Symons TB, Ferrando A, et al. Functional impact of 10 days of bed rest in healthy older adults. J Gerontol A Biol Sci Med Sci 2008, 63:1076-1081.
- Izquierdo M, Aguado X, Gonzalez R, et al. Maximal and explosive force production capacity and balance performance in men of different ages. Eur J Appl Physiol Occup Physiol 1999, 79:260-267.
- Courtney MD, Edwards HE, Chang AM, et al. Improved functional ability and independence in activities of daily living for older adults at high risk of hospital readmission: a randomized controlled trial. J Eval Clin Pract 2012, 18:128-134.
- Fisher SR, Kuo YF, Graham JE, et al. Early ambulation and length of stay in older adults hospitalized for acute illness. Arch Intern Med 2010, 170:1942-1943.
- Cadore EL, Moneo AB, Mensat MM, et al. Positive effects of resistance training in frail elderly patients with dementia after long-term physical restraint. Age (Dordr) 2014, 36:801-811.
- Villareal DT, Chode S, Parimi N, et al. Weight loss, exercise, or both and physical function in obese older adults. N Engl J Med 2011, 364:1218-1229.
- Orwig DL, Hochberg M, Yu-Yahiro J, et al. Delivery and outcomes of a yearlong home exercise program after hip fracture: a randomized controlled trial. Arch Intern Med 2011, 171:323-331.
- Mudge AM, Giebel AJ, Cutler AJ. Exercising body and mind: an integrated approach to functional independence in hospitalized older people. J Am Geriatr Soc 2008, 56:630-635.
- Baztan JJ, Galvez CP, Socorro A. Recovery of functional impairment after acute illness and mortality: one-year follow-up study. Gerontology 2009, 55:269-274.

**HYPOTHESIS**

H1. Functional decline in elderly patients hospitalized for a medical condition can be reversed by implementing a multicomponent exercise program tailored to each patient's clinical circumstances.

H4. A multicomponent exercise program can reduce or prevent cognitive decline in hospitalized elderly patients.

**OBJECTIVES**

General objective:

To analyze whether a multicomponent training program intervention in people aged 75 years or older hospitalized for a medical condition improves functional capacity.

Specific objectives:

1. To analyze the patient subgroups that benefit most from the intervention (frail, disabled, cognitively impaired, malnourished patients, etc.), identifying patient factors (sociodemographic, clinical, functional, and cognitive) and intervention factors (exercise type, intensity, frequency) that could explain differences in the intervention's effectiveness.

2. To analyze whether a multicomponent training program intervention in people aged 75 years or older hospitalized for a medical condition improves cognitive status during follow-up and after hospital discharge.

3. To analyze whether a multicomponent exercise program intervention in people aged 75 years or older hospitalized for a medical condition reduces drug use and iatrogenic effects.

4. To monitor the changes achieved through the intervention over time, as well as the factors that determine the continuation of the benefits of exercise in the short and medium term.

5. To analyze the costs (comparing average length of stay, readmissions, institutionalization, and use of healthcare resources) of the population that exercises compared to the population that does not.

6. To examine the effect of a strength, balance, and gait training program in hospitalized patients on muscle mass, balance ability, muscle power, and gait speed in a clinical setting.

7. To analyze whether a multicomponent exercise program intervention in people aged 75 years or older hospitalized for a medical condition improves the quality of life of participants. 8. To analyze whether a multicomponent training program intervention in people aged 75 years or older hospitalized for a medical condition improves participants' sleep quality.

9. To analyze whether a multicomponent training program intervention in people aged 75 years or older hospitalized for a medical condition produces changes at the omics level.

**Design, study subjects, variables, data collection and analysis, and study limitations.**

Study Design

The project is based on a randomized clinical trial conducted in different European Geriatrics and Internal Medicine Departments that treat hospitalized patients. Patients who meet the inclusion criteria will be randomly assigned to the intervention or control group. Recruitment of hospitalized patients will begin within the first 48 hours of admission to the ward, once the attending physician deems the patient's clinical condition admissible for participation in the program. Patients will be identified from the list of patients admitted to the hospital and assigned to the Geriatrics or Internal Medicine Department. The person who decides on inclusion in the intervention or control group will not be the attending physician. Patients or their caregivers/legal guardians (in the case of patients with cognitive impairment) will be informed of their random inclusion in one of the groups, but will not be informed which one they belong to. Randomization will be performed using the application available at http://www.randomizer.org/. Data will be collected from both the intervention and control groups at four different time points: screening, at hospital discharge, at one month, and at three months.

Study Subjects

People aged 75 years or older hospitalized in European Geriatrics and Internal Medicine departments (HUN, CHU-T, ULSBM, and HNSM).

Inclusion criteria: - Age 75 years or older, admitted to the acute care unit for a medical condition.

Exclusion criteria:- Refusal to sign informed consent by the patient/primary caregiver/legal guardian or inability to obtain it.- Life expectancy of less than 3 months or terminal oncological or non-oncological disease - Inability to follow up - Inability to participate in a multicomponent exercise program - Medical contraindication to exercise - Major neurocognitive disorder moderate and severe stages GDS - Fast Reisberg 5-7 Moderate-severe disability (measured by the Barthel index (BI <60)) - Expected admission of less than 6 days.

Assuming an alpha error of α = 5%, a correlation between pre- and postintervention Short Physical Performance Battery (SPPB) scores of ρ = 0.5, and a standard deviation for the SPPB of σ = 2.5, the sample size required to achieve 90% power to detect a minimum one-point difference between groups in the postintervention SPPB score is 102 patients per group. Taking into account the expected loss of patients at follow-up of 30%, the final sample size required is 148 per group, a total of 296 patients, which implies the inclusion of 37 patients per group in each of the 4 hospitals.

Variables recogidas

1. Variables Independientes:

1.1 Relativas al paciente:

a. Se recogerá la información relativa a la edad y sexo del paciente:

b. Situación funcional previa, durante el ingreso, al alta, al mes y a los 3 meses. Refleja la capacidad del anciano para realizar las actividades de la vida diaria. Se medirá a través del Índice de Barthel.

c. Función física: Se medirá mediante la batería Short Physical Performance Battery (SPPB).

d- Criterios de fragilidad. Se utilizará el fenotipo de fragilidad de Linda Fried.

e. Función cognitiva: Se medirá mediante la prueba Mini Mental State Evaluation (MMSE) y Trail Making Test A para evaluar función ejecutiva.

f. Situación cognitiva previa y síntomas psicoconductuales asociados: Si diagnóstico previo de deterioro cognitivo, estadiaje mediante la escala GDS de Reisberg. Evaluación de síntomas psicoconductuales asociados a demencia. Se evaluará mediante anamnesis su existencia o su ausencia.

g. Delirium mediante la escala 4AT al ingreso

h. Situación anímica: Se evaluará mediante la escala abreviada de depresión GDS-15 de Yesavage.

i. Situación nutricional: Parámetros antropométricos (IMC) y MNA-SF.

j. Calidad de vida: Evalúa el bienestar social general de los individuos. Por su fácil administración, fiabilidad y validez, el EuroQol-5D (EQ-5D).

k. Polifarmacia y psicofármacos: se registrará número de fármacos (se entenderá por polifarmacia el consumo de más de 5 o más fármacos) y número y tipo de psicofármacos y retirada de fármacos.

l. Comorbilidad: Se medirá mediante el índice Cumulative Illness Rating Scale- Geriatric (CIRS-G).

m. Estilos de vida previos: Se recogerán antecedentes personales sobre actividad física (4h de actividad moderad-intensa/ semana); alimentación (raciones semanales fruta-verdura y lácteos); tabaco (sí/no); abuso de alcohol y drogas (sí/no).

n. Caídas, se registrará la existencia y frecuencia de caídas en el último año

o. Antecedentes personales y otros síndromes geriátricos. Enfermedades consideradas agrupadas por códigos CIE-10 Y por códigos de ACG de Salisbury.

p. la calidad del sueño, mediante la escala SATED al alta, al mes y a los 3 meses.

q. Sarcopenia: se evaluará mediante el cuestionario SARC-F durante el ingreso.

1.2. Relativas a la intervención:

a. Fuerza de la extremidad inferior y superior: Se medirá la fuerza de empuje de piernas, empuje pectoral, extensión de rodilla y prensión de manos.

Muestras sanguíneas: Se tomarán muestras sanguíneas 10-20 mL previo ayuno de 10-12 h al inicio y luego de 4 meses de intervención en tubos vacutainer, con EDTA, citrato o secos según el examen requerido. Después de dejar las muestras en posición vertical por 10 minutos, los tubos se centrifugarán a 3000 RPM durante 15 minutos para separar el suero y plasma que serán utilizados para la realización de las determinaciones pertinentes. Parte de estos sueros serán almacenados en viales a 80°C, para posteriormente realizar determinaciones especiales descritas a continuación:

Estudio de Proteómica diferencial mediante Arrays de citoquinas: Este proceso se llevará acabo de acuerdo con la experiencia previa del grupo de investigación de la Unidad de Proteómica Navarrabiomed siguiendo las recomendaciones de la Metabolomics Society (http://www.metabolomicssociety.org/) y la European Nutrigenomics Organisation (NuGO) (http://www.nugo.org/metabolomics). El estudio del proteóma (cluster de inflamación) en sangre se realizará mediante cromatografía líquida acoplada a espectrometría de masas con detector de tiempo de vuelo (HPLC-q-TOF; Applied Biosystems, AB), y aplicando un programa de alineamiento de picos y extracción de los siguientes marcadores: TNF-α, IL-6, IL-10 por técnica ELISA (Enzyme Linked Inmunoabsorvent Assay). hs-C Reactive Protein, ENA-78, GCSF, GM-CSF, GRO, GRO-alpha, I-309, IL-1alpha, IL-1beta, IL-2, IL-3, IL-4, IL-5, IL-6, IL-7, IL-8, IL-10, IL-12 p40/p70, IL-13, IL-15, IFN-gamma, MCP-1, MCP-2, MCP-3, MCSF, MDC, MIG, MIP-1beta, MIP-1delta, RANTES, SCF, SDF-1, TARC, TGF-beta1, TNF-alpha, TNF-beta, EGF, IGF-I, Angiogenin, Oncostatin M, Thrombopoietin, VEGF-A, PDGF-BB, Leptin, BDNF, BLC, Ckß8-1, Eotaxin, Eotaxin-2, Eotaxin-3, FGF-4, FGF-6, FGF-7, FGF-9, Flt-3 Ligand, Fractalkine, GCP-2, GDNF, HGF, IGFBP-1, IGFBP-2, IGFBP-3, IGFBP-4, IL-16, IP-10, LIF, LIGHT, MCP-4, MIF, MIP-3 alpha, NAP-2, NT-3, NT-4, Osteopontin, Osteoprotegerin, PARC, PLGF, TGF-beta2, TGF-beta3, TIMP-1, TIMP-2. Los resultados de este estudio se analizarán con técnicas estadísticas univariantes (ANOVA) y multivariantes (OSC_PLSDA) en los programas informáticos SIMCA-P 11.5 (Umetrics), PermutMatrix 1.9.3 y la plataforma online metaboanalyst. El proteóma se visualizará a través de un gráfico tipo “scores plot” y un gráfico tipo “heat map” y la identificación tentativa de los metabolitos se realizará en bases de datos: KEGG (http://www.genome.jp/), Human Metabolome (www.metabolomics.ca), Metabolome Japan (http://www.metabolome.jp/) MassBank (http://www.massbank.jp/) y NutrimetaboDB (http://www.nugowiki.org/), la cual contiene información acerca de metabolitos del food metaboloma ank.jp/) y NutrimetaboDB (http://www.nugowiki.org/), la cual contiene información acerca de metabolitos del food metaboloma.

2. Outcome variables

a. Changes in functional capacity measured at discharge (in hospitalized patients), at months 1 and 3 compared to baseline for the SPPB and Gait Speed.

b. Difference between the Barthel scores at the initial, final, and 1- and 3-month assessments, as well as percentage of functional recovery.

c. Changes in cognitive variables at months 1 and 3 compared to baseline: MMSE, presence/absence of psychobehavioral symptoms, and TMT-A.

d. Changes in mood at months 1 and 3 compared to baseline: Yesavage GDS.

e. Changes in the number of medications and psychotropic drugs at months 1 and 3 compared to baseline.

f. Falls at one month and three months.

g. Institutionalization at one month and three months.

h. Mortality at 1 month and 3 months.

i. Hospital readmissions at 1 month and 3 months.

j. Changes in quality of life at 1 month and 3 months: Changes in EuroQoL 5D.

k. Changes from baseline to 3 months in maximum isometric strength (HandGrip).

l. Changes from baseline to 3 months in maximum dynamic strength in the lower and upper limbs (1-RM test with strength machines).

m. Average hospital stay and readmission rate.

n. Change in sleep quality (SATED).

Intervention and study subjects

The 74 patients from the HUN Geriatrics Service will be randomly assigned to 2 groups. These groups are:

G0 = Control group (n=37);

G1 = Intervention group: Multicomponent physical training (n=37);

As mentioned above, patients in the control group (G0) will receive the usual care currently provided to other patients, including physical therapy consultation if needed.

The multicomponent exercise intervention group (G1) will be a multicomponent exercise training program, including progressive and supervised aerobic endurance, strength, and balance training lasting 4-7 days during their hospitalization, supervised by the Geriatrics Unit research team. They will also receive individualized guidelines for multicomponent exercise for the first 3 months after hospital discharge.

The multicomponent exercise training program will consist of chair squat exercises. The main part of the training will involve strength training machines for the lower extremities (leg press and knee extension) and one exercise for the upper extremities (seated chest press). The goal is to perform 2-3 sets of 8-12 repetitions at an intensity of 40-70% of the 1 repetition maximum (1RM) (Matrix, Johnson Health Tech, Ibérica, SL, Madrid, Spain), tailored to each individual's functional capacity. Each training session will also include a hip abduction exercise, and finally, balance and stretching exercises will be performed.

Data Analysis

Initially, a descriptive study will be conducted for continuous variables, calculating central tendency and dispersion statistics according to their distribution (mean, standard deviation, and confidence intervals or median and interquartile range) and frequencies for qualitative variables with their respective confidence intervals for the total prevalence. The variable "difference between the initial and final situation" will be created for each patient and for each of the dependent variables, with the objective of defining the size of the change or therapeutic effect. All continuous variables will be tested for a possible normal distribution using the K-S test, using parametric tests when this test passes. In addition, linear regression techniques and Pearson's correlation coefficient will be used to estimate the relationship between quantitative variables, and the Student t test, the Mann-Whitney U test, or analysis of variance (ANOVA) will be used to study the relationship between the means of quantitative variables. The chi-square test and Fisher's exact test will be used to study the relationship between qualitative variables. A multivariate analysis using multiple or logistic regression, as appropriate, will be performed to determine the adjusted risk of presenting the outcome variables. To determine the effectiveness of the intervention, the relative risk with its 95% confidence interval will be calculated. A level of 0.05 will be set to determine statistical significance. The data will be analyzed using SPSS 21.0.

Limitations

One of the main limitations of randomized clinical trials to date is the assessment by subjects blinded to the intervention, as the professionals who are treating the patients at the same time are usually the ones who measure the variables. Furthermore, being such a complex population with significant functional and cognitive impairment, it also makes it difficult to correctly implement and monitor the results. However, this population has routinely been excluded from clinical trials for these reasons, making their inclusion in study groups even more important in order to assess changes in their care patterns. Although the conditions under which patients are recruited and the short duration of the intervention favor the availability of follow-up information, it is necessary to consider the potential loss of individuals inherent in longitudinal studies. The high number of individuals seen annually by the Geriatrics and Internal Medicine clinics of the two centers participating in the study should guarantee a sufficient sample size for this project, despite the aforementioned limitation.

Another important limitation is the fact that the degree of program adherence of patients assigned to the home intervention group cannot be precisely controlled. Home exercise programs are known to pose adherence problems. To minimize this problem, materials will be provided to record the number of weekly training sessions completed. Patients who cannot attend their referral hospital for the 1-month and 3-month assessments will be followed up by telephone at the same time as these assessments. They will be questioned about their adherence to training and will be given scales that can be administered by telephone in order to conduct a realistic analysis.
