# Supplementary material for: Prevention of functional and cognitive impairment through a multicomponent exercise program during and after hospitalization of older adults (PREDISC): Study protocol for a multicenter randomized clinical trial
Source: PLoS One. 2025 Sep 25;20(9):e0332391. doi: 10.1371/journal.pone.0332391 (PMC12463233; doi:10.1371/journal.pone.0332391)
Supplement: S3 Doc — (DOCX) [file pone.0332391.s003.docx]

V1 – 10/05/2024



**PREDISC**

**Prevención del deterioro funcional y cognitivo mediante un programa de ejercicio multicomponente en ancianos hospitalizados (Geriatría y Medicina Interna): ensayo clínico aleatorizado. Estudio multicéntrico (HUN, CHU-T, ULSBM y HNSM)**

Servicio de Geriatría HUN

INGEA - Investigación en Geriatría y Envejecimiento Activo - Navarrabiomed

Universidad Pública de Navarra

INVESTIGADORES:

**HOSPITAL UNIVERSITARIO DE NAVARRA(HUN)-NAVARRABIOMED-UPNA, Navarra**

**Nicolás Martínez Velilla**

Servicio de Geriatría

Hospital Universitario de Navarra

Navarrabiomed – UPNA - IdiSNA

**Fabricio Zambom Ferraresi**

Navarrabiomed – UPNA – IdiSNA

**Iciar Echeverría Beistegui**

Navarrabiomed

**Fabiola Zambom Ferraresi**

Navarrabiomed

**Marisa Fernández Gonzáles de la Riva**

Navarrabiomed

**Maite Izco Cubero**

Navarrabiomed

COLABORADORES:

**CENTRE HOSPITALIER UNIVERSITAIRE DE TOULOUSE POLE GERIATRIE (CHU-T), Occitanie**

**Gabor Abellan Eva Peyrusque**

**Cécile Dedieu**

**UNIDADE LOCAL DE SAUDE BAIXO MONDEGO (ULSBM), Coimbra**

**A. Isabel Gomes Ana Isabel Pais**

**Maria do Rosário Pereira Costa Santos Silva**

**HOSPITAL NOSTRA SENYORA DE MERITXELL (HNSM), Servei Envelliment i Salut del SAAS, Andorra**

**Eva Heras Jan Missé**

**Encarna Ulloa Maria Anglada**

**RESUMEN DEL PROYECTO DE INVESTIGACIÓN**

| PROMOTOR | Navarrabiomed. Hospital Universitario de Navarra. Universidad Pública de Navarra |
| --- | --- |
| DIRECTOR Y SUPERVISOR  DEL ESTUDIO DE INVESTIGACIÓN | Nicolás Martínez Velilla |
| TITULO | **PREDISC**: Prevención del deterioro funcional y cognitivo mediante un programa de ejercicio multicomponente en ancianos hospitalizados (Geriatría y Medicina Interna): ensayo clínico aleatorizado. Estudio multicéntrico (HUN, CHU-T, ULSBM y HNSM). |
| OBJETIVOS | Objetivo general: Analizar si una intervención mediante un programa de entrenamiento multicomponente en personas con edad igual o superior a 75 años hospitalizadas por patología médica mejora la capacidad funcional.  Objetivos específicos:  1. Analizar los subgrupos de pacientes que más se benefician de la intervención (pacientes frágiles, discapacitados, con deterioro cognitivo, con desnutrición, etc.) identificando factores del paciente (sociodemográficos, clínicos, funcionales y cognoscitivos) y de la intervención (tipo de ejercicio, intensidad, frecuencia), que podrían explicar las diferencias en la efectividad de la intervención.  2. Analizar si una intervención mediante un programa de entrenamiento multicomponente en personas con edad igual o superior a 75 años hospitalizados por enfermedad médica mejora el estado cognitivo durante su seguimiento y tras el alta hospitalaria.  3. Analizar si una intervención mediante un programa de ejercicio físico multicomponente en personas con edad igual o superior a 75 años hospitalizados por enfermedad médica disminuye el consumo de fármacos y la iatrogenia farmacológica.  4. Monitorizar evolutivamente las modificaciones alcanzadas mediante la intervención, así como los factores que determinan la perpetuación de los beneficios del ejercicio a corto y medio plazo.  5. Analizar los costes (comparar estancias medias, reingresos, institucionalización y uso de recursos sanitarios) de la población que realiza ejercicio respecto a la población que no lo realiza.  6. Examinar el efecto de un programa de entrenamiento de fuerza, equilibrio y marcha en pacientes hospitalizados sobre la masa muscular, la capacidad de equilibrio, la potencia muscular y la velocidad de marcha en el ámbito clínico.  7. Analizar si una intervención mediante un programa de entrenamiento multicomponente en personas con edad igual o superior a 75 años hospitalizados por enfermedad médica mejora la calidad de vida de los participantes.  8. Analizar si una intervención mediante un programa de entrenamiento multicomponente en personas con edad igual o superior a 75 años hospitalizados por enfermedad médica mejora la calidad del sueño de los participantes.  9. Analizar si una intervención mediante un programa de entrenamiento multicomponente en personas con edad igual o superior a 75 años hospitalizados por enfermedad médica produce cambios a nivel de Ómicas. |
| DISEÑO DEL ESTUDIO | Ensayo Clínico Aleatorizado - Multicéntrico |
| CRITERIOS DE INCLUSIÓN | - Personas con edad >75 años hospitalizadas en servicios de Geriatría y Medicina Interna europeos (HUN, CHU-T, ULSBM y HNSM).  - Capaz de comunicarse.  - Capaz de deambular, con o sin asistencia personal / técnica o moverse sin ayuda en una silla de ruedas. |
| CRITERIOS DE EXCLUSIÓN | - Negativa a firmar el consentimiento informado por parte del paciente/cuidador principal /tutor legal o imposibilidad de obtenerlo.  - Expectativa de vida inferior a 3 meses o enfermedad oncológica o no oncológica en fase terminal.  - Imposibilidad de seguimiento.  - Imposibilidad para participar en un programa de ejercicio multicomponente.  - Contraindicación médica para realizar ejercicio.  - Trastorno neurocognitivo estadio moderado y severo GDS - Fast Reisberg 5-7  - Discapacidad moderada-severa (medida por el índice de Barthel (IB <60).  - Previsión de ingreso inferior a 6 días. |
| TAMAÑO MUESTRAL | 296 pacientes entre los 4 centros participantes – 74 pacientes en Navarra (37 grupo control y 37 grupo intervención) |
| DURACIÓN DEL ESTUDIO | 30 meses |

**INTRODUCCIÓN**

El progresivo envejecimiento de la sociedad y el reto que supone la atención al anciano se ha convertido en una auténtica urgencia para todos los países y sistemas de salud. Los ancianos protagonizan de manera creciente la actividad de los servicios sanitarios y servicios sociales, y sus necesidades son totalmente distintas a la población adulta y a las que se planteaban 50 años atrás. En este sentido uno de los principales organismos sanitarios mundiales, la Organización Mundial de la Salud (OMS), ha publicado recientemente el primer "Informe mundial sobre envejecimiento y salud (“World Report on Ageing and Health”). Una de las mayores novedades del informe es la aparición del concepto **capacidad intrínseca**, entendida como el conjunto de las capacidades físicas y mentales que dota al individuo de habilidades funcionales que le permitan afrontar los retos que el entorno genera. Uno de los mensajes fundamentales del informe es que los sistemas de salud que atiendan ancianos deberían sustituir su foco de atención sobre la enfermedad y centrarlo en la capacidad intrínseca (es decir **la función**). Esto es particularmente importante en la atención a los ancianos vulnerables que están en riesgo de discapacidad (los frágiles y con deterioro en la cognición), para poder actuar precozmente sobre ellos.

La **fragilidad** se puede definir como una condición clínica en la cual existe un riesgo incrementado en la vulnerabilidad individual para desarrollar eventos adversos como dependencia y/o mortalidad cuando hay exposición a estresores. Un consenso internacional reciente define la fragilidad física como un importante «síndrome médico con múltiples causas y contribuyentes que se caracteriza por una disminución de fuerza, resistencia y función fisiológica que incrementa la vulnerabilidad individual para desarrollar mayor dependencia y/o muerte». La prevalencia de este síndrome es alta en mayores de 65 años, dependiendo del método de cribado utilizado, (7-16%) y aumenta con la edad. El consenso más establecido sobre el fenotipo diagnóstico engloba varios dominios, incluyendo el deterioro en la función física (velocidad de la marcha enlentecida, fatiga, fuerza de prensión disminuida), pérdida de peso y una baja actividad física. Vinculado al fenómeno del envejecimiento, la capacidad funcional del sistema neuromuscular, cardiovascular y respiratorio comienza a disminuir de forma progresiva lo que conlleva un riesgo aumentado de fragilidad.

El **abordaje tradicional del concepto de fragilidad** ha partido de un fenotipo clínico, pero de forma progresiva se han ido incorporando conocimientos a nivel bioquímico y molecular. Por ejemplo, es importante destacar la relación entre los niveles bajos de IGF-1 con la sarcopenia y la discapacidad, así como la relación inversamente proporcional de factores inflamatorios como por ejemplo la interleucina 6 y su relación con la proteólisis muscular, discapacidad y mortalidad. Por otro lado, aunque con evidencias crecientes del rol de la microbiota en los organismos, no se ha realizado hasta el momento una vinculación con la vulnerabilidad de los ancianos. La integridad del epitelio intestinal protege al organismo de las agresiones. Una lesión o pérdida de la barrera intestinal puede desencadenar el desarrollo de diferentes tipos de enfermedades inflamatorias intestinales y de algunas enfermedades autoinmunes. Hasta el momento existe una limitada información relacionada a la modificación de la microbiota en situaciones de hospitalización, así como los efectos protectores que pueda tener un programa de ejercicio físico multicomponente.

El **paciente anciano hospitalizado** está condicionando por una serie de circunstancias ajenas al propio proceso patológico que causó el ingreso hospitalario y que habitualmente empeoran los resultados de la hospitalización. De hecho, la implantación de **modelos asistenciales** diferentes a los **tradicionales** ha mostrado un claro beneficio en los resultados funcionales de estos pacientes a corto y medio plazo. No obstante, los componentes de estos modelos que explican estos mejores resultados no han sido evaluados de manera diferencial. Algunos ejemplos de estas circunstancias son las habituales ordenes de reposo absoluto sin ningún criterio objetivo para pacientes que son capaces de deambular, la perpetuación de la sueroterapia continua, las restricciones físicas y químicas, sondajes permanentes innecesarios, etc. En numerosas ocasiones los pacientes ancianos hospitalizados pasan la mayor parte del tiempo encamados llegando a ser incluso superior al 83% de encamamiento frente a un 4% de los que se ponen de pie o están paseando. Esta población, al tener reducida su reserva funcional y fisiológica son más vulnerables a los efectos por ejemplo del encamamiento y que abarcan desde la pérdida funcional o el deterioro cognitivo, a las mayores estancias, mortalidad e institucionalización, peor situación anímica, delirium, desacondicionamiento, aspiraciones, úlceras por presión y caídas, disminución de la ingesta calórica, aislamiento social, peor calidad de vida y mayor uso de recursos relacionados con la salud.

Los beneficios del **ejercicio físico** en el envejecimiento y específicamente en la fragilidad han sido objeto de reciente investigación científica. Así, se ha comprobado como una actividad física incrementada en el anciano se ha asociado con una disminución del riesgo de mortalidad, del riesgo de enfermedades crónicas prevalentes en el envejecimiento (cardiovasculares, osteoarticulares, neurodegenerativas) institucionalización, y de deterioro funcional. De manera más concreta, el tipo de ejercicio físico más beneficioso en el anciano frágil es el denominado multicomponente. Este tipo de programas combina entrenamiento de fuerza, resistencia, equilibrio y marcha, y es el que ha demostrado mejorías más significativas en la capacidad funcional, que es un elemento fundamental para el mantenimiento de la independencia en las actividades básicas e instrumentales de la vida diaria de los ancianos.

Dada la relación comentada entre **fragilidad y deterioro cognitivo**, tiene sentido que aquellas intervenciones que resultaran eficaces en el paciente frágil pudiesen ser beneficiosas en el anciano con deterioro cognitivo y viceversa. Aunque los estudios son escasos, en algunos recientes se demuestra cómo programas de ejercicio de resistencia semanales durante 12 semanas, en una cohorte de ancianas, no solo provocan aumentos de la velocidad de la marcha, sino que resultan beneficiosos en la mejoría de funciones cognitivas ejecutivas, que están interesantemente relacionadas con el riesgo de caída.

En nuestro grupo de investigación hemos corroborado estos resultados realizado intervenciones con programas de ejercicio multicomponente en ancianos institucionalizados frágiles con deterioro cognitivo leve o demencia con mejoras significativas en la capacidad funcional, riesgo de caídas y funciones cognitivas ejecutivas. Otros autores han encontrado resultados similares en cuanto a reversión de la fragilidad y mejoras cognitivas, aunque suelen excluirse de forma basal a los pacientes con deterioro cognitivo inicial, dificultado la extrapolación de los resultados a un porcentaje importante de ancianos.

Sin embargo, a pesar de la abrumadora evidencia científica que la actividad física mejora la salud de la población, la sociedad en general y los profesionales sanitarios en particular, todavía no se acaba de implementar en nuestros sistemas sanitarios. Esto implicaría necesariamente una **prescripción individualizada** acorde a la capacidad funcional del anciano con recomendaciones específicas sobre las dosis (intensidad, volumen, frecuencia) tal y como se hace con el resto de los medicamentos, con la ventaja de que la iatrogenia subyacente a los fármacos no existe con el ejercicio.

El programa europeo de prescripción de ejercicio físico **VIVIFRAIL** (www.vivifrail.com) que se aplicará en este estudio y que ha sido desarrollado por expertos a nivel mundial del ámbito de la actividad física y de la fragilidad (y que forman parte del equipo investigador, entre ellos el coordinador del programa), consideramos que supone un hito en este sentido. Por primera vez se avanza de forma significativa en la mencionada "**receta de ejercicio físico**" individualizada y su aplicación a una población tan vulnerable como los ancianos frágiles y con problemas cognitivos. Apostamos que puede suponer un avance significativo en la mejora de la capacidad funcional de estos pacientes y que como dice la OMS, debe ser el objetivo de los sistemas de salud que atienden a estas personas.

Si nuestra hipótesis es correcta se abre la puerta a **modificación del sistema de hospitalización** y **manejo ambulatorio** actual a pacientes ancianos. Si modificamos las pautas actuales es probable que los ancianos después del ingreso presenten menores **niveles de deterioro funcional y cognitivo**, y previsiblemente una mejor **calidad de vida** y un menor **consumo de recursos sanitarios** (menores reingresos, menor institucionalización, entre otros). Por otro lado, mientras que habitualmente las intervenciones de ejercicio en pacientes de edad avanzada se han realizado a participantes en la comunidad, instituciones u hospitalizados para rehabilitación, existe falta de evidencia en pacientes de edad avanzada con patología médica aguda, que con frecuencia presentan comorbilidades múltiples.

Un aspecto muy importante de nuestro estudio es que los únicos criterios de exclusión (a diferencia de la mayoría de los estudios previos) permiten la participación de ancianos con diferentes niveles cognitivos y funcionales, pues la premisa única es que puedan deambular y participar activamente en el programa.

Referencias:

- Martínez-Velilla N, Casas-Herrero A, Zambom-Ferraresi F, Sáez de Asteasu ML, Lucia A, Galbete A, García-Baztán A, Alonso-Renedo J, González-Glaría B, Gonzalo-Lázaro M, Apezteguía Iráizoz I, Gutiérrez-Valencia M, Rodríguez-Mañas L, Izquierdo M. Effect of Exercise Intervention on Functional Decline in Very Elderly Patients During Acute Hospitalization: A Randomized Clinical Trial. JAMA Intern Med (IF: 21.87; Q1). 2019 Jan 1;179(1):28-36. doi: 10.1001/jamainternmed.2018.4869. PMID: 30419096
- Martínez-Velilla N, Abizanda P, Gómez-Pavón J, Zambom-Ferraresi F, Sáez de Asteasu ML, Fiatarone Singh M, Izquierdo M. Effect of an Exercise Intervention on Functional Decline in Very Old Patients During Acute Hospitalizations: Results of a Multicenter, Randomized Clinical Trial. JAMA Intern Med (IF: 21.87; Q1). 2022 Mar 1;182(3):345-347. doi: 10.1001/jamainternmed.2021.7654. PMID: 35040873
- Word Health Organization (2015). World Report on Aging on Ageing. Geneva. ISBN: 978 9241565042
- Clegg A, Joung G, Lille S et al. Frailty in elderly people. Lancet 2013: 381: 752-762
- Rodriguez Mañas L., Fried L. Frailty in the clinical scenario. Lancet 2015; 385: e7-e9.
- Robertson DA, Savva GM, Coen RF, Kenny RA. Cognitive function in the prefrailty and frailty syndrome. J Am Geriatr Soc. 2014 Nov;62(11):2118-24.
- Cognitive frailty: rational and definition from an (I.A.N.A./I.A.G.G.) international consensus group.
- Kelaiditi E, Cesari M, Canevelli M, van Kan GA, Ousset PJ, Gillette-Guyonnet S et al ; IANA/IAGG.J Nutr Health Aging. 2013 Sep;17(9):726-34
- Liu-AmBrose t, NagaMatsuls, GraF P, Beattie Bl, asHe MC, HanDytC. Resistance training and executive functions: a 12-month randomized controlled trial. ArchInternMed 2010 25; 170: 170-178.
- Zheng G1, Xia R2, Zhou W2, Tao J2, Chen L3. Aerobic exercise ameliorates cognitive function in older adults with mild cognitive impairment: a systematic review and meta-analysis of randomised controlled trials. Br J SportsMed. 2016 Apr 19.
- Cadore EL, Moneo AB, Mensat MM, Muñoz AR, Casas-Herrero A, Rodriguez-Mañas L, Izquierdo M. Positive effects of resistance training in frail elderly patients with dementia after long-term physical restraint. Age (Dordr). 2014 Apr;36(2):801-11.
- Casas-Herrero A, Cadore EL, Zambom-Ferraresi F, Idoate F, Millor N, Martínez-Ramirez A, Gómez M, Rodriguez-Mañas L, Marcellán T, de GordoaAR,Marques MC, Izquierdo M. Functional capacity, muscle fat infiltration, power output, and cognitive impairment in institutionalized frail oldes told. Rejuvenation Res. 2013 Oct;16(5):396-403.
- Tarazona-Santabalbina FJ, Gómez-Cabrera MC, Pérez-Ros P, Martínez-Arnau FM, Cabo H,Tsaparaset al A Multicomponent Exercise Intervention that Reverses Frailty and Improves Cognition, Emotion, and Social Networking in the Community-Dwelling Frail Elderly: A Randomized Clinical Trial. Am MedDirAssoc. 2016 May 1;17(5):426-33.
- Volpato S, Onder G, Cavalieri M, et al. Characteristics of nondisabled older patients developing new disability associated with medical illnesses and hospitalization. J Gen Intern Med 2007, 22:668-674.
- Baztan JJ, Suarez-Garcia FM, Lopez-Arrieta J, et al. Effectiveness of geriatric units on functional decline, living at home, and case fatality among older patients admitted to hospital for acute medical disorders: meta-analysis. BMJ 2009, 338: b50.
- Covinsky KE, Palmer RM, Fortinsky RH, et al. Loss of independence in activities of daily living in older adults hospitalized with medical illnesses: increased vulnerability with age. J Am Geriatr Soc 2003, 51:451-458.
- Brown CJ, Redden DT, Flood KL, et al. The underrecognized epidemic of low mobility during hospitalization of older adults. J Am Geriatr Soc 2009, 57:1660-1665.
- Brown CJ, Friedkin RJ, Inouye SK. Prevalence and outcomes of low mobility in hospitalized older patients. J Am Geriatr Soc 2004, 52:1263-1270.
- Creditor MC. Hazards of hospitalization of the elderly. Ann Intern Med 1993, 118:219-223.
- Allman RM, Goode PS, Patrick MM, et al. Pressure ulcer risk factors among hospitalized patients with activity limitation. JAMA 1995, 273:865-870.
- Gillick MR, Serrell NA, Gillick LS. Adverse consequences of hospitalization in the elderly. Soc Sci Med 1982, 16:1033-1038.
- Fortinsky RH, Covinsky KE, Palmer RM, et al. Effects of functional status changes before and during hospitalization on nursing home admission of older adults. J Gerontol A Biol Sci Med Sci 1999, 54:M521-526.
- Narain P, Rubenstein LZ, Wieland GD, et al. Predictors of immediate and 6-month outcomes in hospitalized elderly patients. The importance of functional status. J Am Geriatr Soc 1988, 36:775-783.
- Hirsch CH, Sommers L, Olsen A, et al. The natural history of functional morbidity in hospitalized older patients. J Am Geriatr Soc 1990, 38:1296-1303.
- Inouye SK, Wagner DR, Acampora D, et al. A predictive index for functional decline in hospitalized elderly medical patients. J Gen Intern Med 1993, 8:645-652.
- Sager MA, Franke T, Inouye SK, et al. Functional outcomes of acute medical illness and hospitalization in older persons. Arch Intern Med 1996, 156:645-652.
- Sager MA, Rudberg MA, Jalaluddin M, et al. Hospital admission risk profile (HARP): identifying older patients at risk for functional decline following acute medical illness and hospitalization. J Am Geriatr Soc 1996, 44:251-257.
- Rudberg MA, Sager MA, Zhang J. Risk factors for nursing home use after hospitalization for medical illness. J Gerontol A Biol Sci Med Sci 1996, 51:M189-194.
- Covinsky KE, Justice AC, Rosenthal GE, et al. Measuring prognosis and case mix in hospitalized elders. The importance of functional status. J Gen Intern Med 1997, 12:203-208.
- Lakhan P, Jones M, Wilson A, et al. A prospective cohort study of geriatric syndromes among older medical patients admitted to acute care hospitals. J Am Geriatr Soc 2011, 59:2001-2008.
- Hannan EL, Magaziner J, Wang JJ, et al. Mortality and locomotion 6 months after hospitalization for hip fracture: risk factors and risk-adjusted hospital outcomes. JAMA 2001, 285:2736-2742.
- Kortebein P, Symons TB, Ferrando A, et al. Functional impact of 10 days of bed rest in healthy older adults. J Gerontol A Biol Sci Med Sci 2008, 63:1076-1081.
- Izquierdo M, Aguado X, Gonzalez R, et al. Maximal and explosive force production capacity and balance performance in men of different ages. Eur J Appl Physiol Occup Physiol 1999, 79:260-267.
- Courtney MD, Edwards HE, Chang AM, et al. Improved functional ability and independence in activities of daily living for older adults at high risk of hospital readmission: a randomized controlled trial. J Eval Clin Pract 2012, 18:128-134.
- Fisher SR, Kuo YF, Graham JE, et al. Early ambulation and length of stay in older adults hospitalized for acute illness. Arch Intern Med 2010, 170:1942-1943.
- Cadore EL, Moneo AB, Mensat MM, et al. Positive effects of resistance training in frail elderly patients with dementia after long-term physical restraint. Age (Dordr) 2014, 36:801-811.
- Villareal DT, Chode S, Parimi N, et al. Weight loss, exercise, or both and physical function in obese older adults. N Engl J Med 2011, 364:1218-1229.
- Orwig DL, Hochberg M, Yu-Yahiro J, et al. Delivery and outcomes of a yearlong home exercise program after hip fracture: a randomized controlled trial. Arch Intern Med 2011, 171:323-331.
- Mudge AM, Giebel AJ, Cutler AJ. Exercising body and mind: an integrated approach to functional independence in hospitalized older people. J Am Geriatr Soc 2008, 56:630-635.
- Baztan JJ, Galvez CP, Socorro A. Recovery of functional impairment after acute illness and mortality: one-year follow-up study. Gerontology 2009, 55:269-274.

**HIPÓTESIS**

H1. El deterioro funcional en pacientes ancianos hospitalizados por patología médica es susceptible de ser revertido mediante la realización de un programa de ejercicio físico multicomponente adaptado a las circunstancias clínicas de cada paciente.

H4. Un programa de ejercicio multicomponente puede reducir o prevenir el deterioro cognitivo en ancianos hospitalizados.

**OBJETIVOS**

Objetivo general:

Analizar si una intervención mediante un programa de entrenamiento multicomponente en personas con edad igual o superior a 75 años hospitalizadas por patología médica mejora la capacidad funcional

Objetivos específicos:

1. Analizar los subgrupos de pacientes que más se benefician de la intervención (pacientes frágiles, discapacitados, con deterioro cognitivo, con desnutrición, etc.) identificando factores del paciente (sociodemográficos, clínicos, funcionales y cognoscitivos) y de la intervención (tipo de ejercicio, intensidad, frecuencia), que podrían explicar las diferencias en la efectividad de la intervención.

2. Analizar si una intervención mediante un programa de entrenamiento multicomponente en personas con edad igual o superior a 75 años hospitalizados por enfermedad médica mejora el estado cognitivo durante su seguimiento y tras el alta hospitalaria.

3. Analizar si una intervención mediante un programa de ejercicio físico multicomponente en personas con edad igual o superior a 75 años hospitalizados por enfermedad médica disminuye el consumo de fármacos y la iatrogenia farmacológica.

4. Monitorizar evolutivamente las modificaciones alcanzadas mediante la intervención, así como los factores que determinan la perpetuación de los beneficios del ejercicio a corto y medio plazo.

5. Analizar los costes (comparar estancias medias, reingresos, institucionalización y uso de recursos sanitarios) de la población que realiza ejercicio respecto a la población que no lo realiza.

6. Examinar el efecto de un programa de entrenamiento de fuerza, equilibrio y marcha en pacientes hospitalizados sobre la masa muscular, la capacidad de equilibrio, la potencia muscular y la velocidad de marcha en el ámbito clínico.

7. Analizar si una intervención mediante un programa de entrenamiento multicomponente en personas con edad igual o superior a 75 años hospitalizados por enfermedad médica mejora la calidad de vida de los participantes.

8. Analizar si una intervención mediante un programa de entrenamiento multicomponente en personas con edad igual o superior a 75 años hospitalizados por enfermedad médica mejora la calidad del sueño de los participantes.

9. Analizar si una intervención mediante un programa de entrenamiento multicomponente en personas con edad igual o superior a 75 años hospitalizados por enfermedad médica produce cambios a nivel de Ómicas.

**Diseño, sujetos de estudio, variables, recogida y análisis de datos y limitaciones del estudio.**

Diseño del estudio

El proyecto se basa en un ensayo clínico aleatorizado realizado en diferentes **Servicios de Geriatría y Medicina Interna europeos** que atienden pacientes hospitalizado. Los pacientes que cumplan los criterios de inclusión serán asignados de forma aleatoria al grupo intervención o control. El reclutamiento de los pacientes hospitalizados comenzará en las primeras 48 horas del ingreso en planta, una vez que el médico responsable considere que la situación clínica del paciente le permite participar en el programa. Estos serán identificados a través de la lista de pacientes admitidos en el hospital y asignados al Servicio de Geriatría o Medicina Interna. La persona que decide la inclusión en el grupo de intervención o control no será el médico responsable del paciente. Los pacientes o sus cuidadores/ tutores legales (en el caso de que el paciente tenga deterioro cognitivo) serán informados de la inclusión aleatoria en uno de los grupos, pero no se les informará a cuál pertenecen. La aleatorización se realizará mediante la aplicación disponible en http://www.randomizer.org/. La información, tanto en el grupo intervenido como en el grupo control, se obtendrá en cuatro momentos diferentes: screening, al alta hospitalaria, al mes y a los 3 meses.

Sujetos de estudio

Personas con edad igual o superior a 75 años hospitalizadas en servicios de Geriatría y Medicina Interna europeos **(HUN, CHU-T, ULSBM y HNSM)**.

Criterios de inclusión: - Edad igual o mayor a 75 años, ingresados a la unidad de agudos por patología médica.

Criterios de exclusión:- Negativa a firmar el consentimiento informado por parte del paciente/cuidador principal /tutor legal o imposibilidad de obtenerlo.- Expectativa de vida inferior a 3 meses o enfermedad oncológica o no oncológica en fase terminal - Imposibilidad de seguimiento - Imposibilidad para participar en un programa de ejercicio multicomponente - Contraindicación médica para realizar ejercicio - Trastorno neurocognitivo mayor estadio moderado y severo GDS - Fast Reisberg 5-7 Discapacidad moderada-severa (medida por el índice de Barthel (IB <60) - Previsión de ingreso inferior a 6 días.

Suponiendo un error alfa de α = 5%, una correlación entre los valores previos y posteriores a la intervención de la Short Physical Performance Battery (SPPB) de ρ = 0,5, y una desviación estándar para la SPPB de σ = 2,5, el **tamaño de la muestra** necesario para alcanzar una potencia del 90% para detectar una diferencia mínima de un punto entre los grupos en la puntuación SPPB posterior a la intervención es de 102 pacientes por grupo. Teniendo en cuenta la pérdida esperada de pacientes en un seguimiento del 30%, el tamaño final de la muestra necesaria es de 148 por grupo, un total de 296 pacientes., lo que supone la inclusión de 37 pacientes por grupo en cada uno de los 4 hospitales.

Variables recogidas

1. Variables Independientes:

1.1 Relativas al paciente:

a. Se recogerá la información relativa a la edad y sexo del paciente:

b. Situación funcional previa, durante el ingreso, al alta, al mes y a los 3 meses. Refleja la capacidad del anciano para realizar las actividades de la vida diaria. Se medirá a través del Índice de Barthel.

c. Función física: Se medirá mediante la batería Short Physical Performance Battery (SPPB).

d- Criterios de fragilidad. Se utilizará el fenotipo de fragilidad de Linda Fried.

e. Función cognitiva: Se medirá mediante la prueba Mini Mental State Evaluation (MMSE) y Trail Making Test A para evaluar función ejecutiva.

f. Situación cognitiva previa y síntomas psicoconductuales asociados: Si diagnóstico previo de deterioro cognitivo, estadiaje mediante la escala GDS de Reisberg. Evaluación de síntomas psicoconductuales asociados a demencia. Se evaluará mediante anamnesis su existencia o su ausencia.

g. Delirium mediante la escala 4AT al ingreso

h. Situación anímica: Se evaluará mediante la escala abreviada de depresión GDS-15 de Yesavage.

i. Situación nutricional: Parámetros antropométricos (IMC) y MNA-SF.

j. Calidad de vida: Evalúa el bienestar social general de los individuos. Por su fácil administración, fiabilidad y validez, el EuroQol-5D (EQ-5D).

k. Polifarmacia y psicofármacos: se registrará número de fármacos (se entenderá por polifarmacia el consumo de más de 5 o más fármacos) y número y tipo de psicofármacos y retirada de fármacos.

l. Comorbilidad: Se medirá mediante el índice Cumulative Illness Rating Scale- Geriatric (CIRS-G).

m. Estilos de vida previos: Se recogerán antecedentes personales sobre actividad física (4h de actividad moderad-intensa/ semana); alimentación (raciones semanales fruta-verdura y lácteos); tabaco (sí/no); abuso de alcohol y drogas (sí/no).

n. Caídas, se registrará la existencia y frecuencia de caídas en el último año

o. Antecedentes personales y otros síndromes geriátricos. Enfermedades consideradas agrupadas por códigos CIE-10 Y por códigos de ACG de Salisbury.

p. la calidad del sueño, mediante la escala SATED al alta, al mes y a los 3 meses.

q. Sarcopenia: se evaluará mediante el cuestionario SARC-F durante el ingreso.

1.2. Relativas a la intervención:

a. Fuerza de la extremidad inferior y superior: Se medirá la fuerza de empuje de piernas, empuje pectoral, extensión de rodilla y prensión de manos.

Muestras sanguíneas: Se tomarán muestras sanguíneas 10-20 mL previo ayuno de 10-12 h al inicio y luego de 4 meses de intervención en tubos vacutainer, con EDTA, citrato o secos según el examen requerido. Después de dejar las muestras en posición vertical por 10 minutos, los tubos se centrifugarán a 3000 RPM durante 15 minutos para separar el suero y plasma que serán utilizados para la realización de las determinaciones pertinentes. Parte de estos sueros serán almacenados en viales a 80°C, para posteriormente realizar determinaciones especiales descritas a continuación:

Estudio de Proteómica diferencial mediante Arrays de citoquinas: Este proceso se llevará acabo de acuerdo con la experiencia previa del grupo de investigación de la Unidad de Proteómica Navarrabiomed siguiendo las recomendaciones de la Metabolomics Society (http://www.metabolomicssociety.org/) y la European Nutrigenomics Organisation (NuGO) (http://www.nugo.org/metabolomics). El estudio del proteóma (cluster de inflamación) en sangre se realizará mediante cromatografía líquida acoplada a espectrometría de masas con detector de tiempo de vuelo (HPLC-q-TOF; Applied Biosystems, AB), y aplicando un programa de alineamiento de picos y extracción de los siguientes marcadores: TNF-α, IL-6, IL-10 por técnica ELISA (Enzyme Linked Inmunoabsorvent Assay). hs-C Reactive Protein, ENA-78, GCSF, GM-CSF, GRO, GRO-alpha, I-309, IL-1alpha, IL-1beta, IL-2, IL-3, IL-4, IL-5, IL-6, IL-7, IL-8, IL-10, IL-12 p40/p70, IL-13, IL-15, IFN-gamma, MCP-1, MCP-2, MCP-3, MCSF, MDC, MIG, MIP-1beta, MIP-1delta, RANTES, SCF, SDF-1, TARC, TGF-beta1, TNF-alpha, TNF-beta, EGF, IGF-I, Angiogenin, Oncostatin M, Thrombopoietin, VEGF-A, PDGF-BB, Leptin, BDNF, BLC, Ckß8-1, Eotaxin, Eotaxin-2, Eotaxin-3, FGF-4, FGF-6, FGF-7, FGF-9, Flt-3 Ligand, Fractalkine, GCP-2, GDNF, HGF, IGFBP-1, IGFBP-2, IGFBP-3, IGFBP-4, IL-16, IP-10, LIF, LIGHT, MCP-4, MIF, MIP-3 alpha, NAP-2, NT-3, NT-4, Osteopontin, Osteoprotegerin, PARC, PLGF, TGF-beta2, TGF-beta3, TIMP-1, TIMP-2. Los resultados de este estudio se analizarán con técnicas estadísticas univariantes (ANOVA) y multivariantes (OSC_PLSDA) en los programas informáticos SIMCA-P 11.5 (Umetrics), PermutMatrix 1.9.3 y la plataforma online metaboanalyst. El proteóma se visualizará a través de un gráfico tipo “scores plot” y un gráfico tipo “heat map” y la identificación tentativa de los metabolitos se realizará en bases de datos: KEGG (http://www.genome.jp/), Human Metabolome (www.metabolomics.ca), Metabolome Japan (http://www.metabolome.jp/) MassBank (http://www.massbank.jp/) y NutrimetaboDB (http://www.nugowiki.org/), la cual contiene información acerca de metabolitos del food metaboloma.

2. Variables de resultado

a. Cambios en la capacidad funcional medida al alta (en los hospitalizados), al 1º y 3º mes respecto a la de la valoración basal para el SPPB y la Velocidad de la Marcha.

b. Diferencia entre el Barthel de la valoración inicial, final, al mes y 3 meses, así como porcentaje de recuperación funcional.

c. Cambios en variables cognitivas en los meses 1º y 3º con respecto a la valoración basal: MMSE, existencia/ausencia de síntomas psico-conductuales y TMT-A.

d. Cambios en la situación anímica en los meses 1º y 3º con respecto valoración basal: GDS Yesavage.

e. Cambios en el número de fármacos y psicofármacos en los meses 1º y 3º con respecto valoración basal.

f. Caídas al mes y 3 meses.

g. Institucionalización al mes y 3 meses.

h. Mortalidad al mes y a los 3 meses.

i. Reingresos hospitalarios al mes y a los 3 meses.

j. Cambios en la calidad de vida al mes y 3 meses: Cambios en el EuroQoL 5D.

k. Cambios valoración inicial-3 meses en fuerza máxima isométrica (HandGrip).

l. Cambios valoración inicial- tres meses en fuerza máxima dinámica en miembros inferiores y superiores (test de 1-RM con máquinas de fuerza).

m. Estancia media hospitalaria y tasa de reingresos.

n. Cambio en la calidad del sueño (SATED)

Intervención y sujetos del estudio

Los 74 pacientes del Servicio de Geriatría del HUN serán asignados de forma aleatorizada a los 2 grupos. Siendo ellos:

G0 = Grupo control (n=37);

G1 = Grupo Intervención: Entrenamiento físico multicomponente (n=37);

Como se ha mencionado anteriormente, los pacientes del grupo control (G0) recibirán la atención usual que se proporciona actualmente a los demás pacientes, en que incluye interconsulta a fisioterapia en el caso de que la necesiten.

La intervención del grupo de intervención de ejercicio físico multicomponente (G1) consistirá en un programa de entrenamiento físico multicomponente, que incluirá entrenamiento físico progresivo y supervisado de resistencia aeróbica, fuerza y equilibrio con una duración de 4-7 días durante su hospitalización, y será supervisado por el equipo de investigación de la Unidad de Geriatría. Además, recibirán pautas individualizadas para que hagan ejercicio físico multicomponente durante los 3 meses tras el alta hospitalario.

El programa de entrenamiento físico multicomponente constará de ejercicios de sentadilla en silla. En la parte principal del entrenamiento se utilizarán máquinas para el entrenamiento de fuerza para las extremidades inferiores (prensa de piernas y extensión de rodillas) y un ejercicio para las extremidades superiores (prensa de pecho sentado). El objetivo es realizar 2-3 series de 8-12 repeticiones a una intensidad del 40-70% de 1 repetición máxima (1RM) (Matrix, Johnson Health Tech, Ibérica, SL, Madrid, España), adaptados a la capacidad funcional de cada individuo. Cada sesión de entrenamiento incluirá también un ejercicio de abducción de cadera y, por último, se realizarán ejercicios de equilibrio y estiramientos.

Análisis de datos

Inicialmente se realizará un estudio descriptivo para variables continuas, calculando los estadísticos de tendencia central y dispersión según su distribución (medias, desviación típica e intervalos de confianza o mediana y rango intercuartil) y de frecuencias para variables cualitativas con sus respectivos intervalos de confianza en las prevalencias totales. Se creará la variable “diferencia entre la situación inicial y la situación final” para cada paciente y para cada una de las variables dependientes, con el objetivo de definir el tamaño del cambio o efecto terapéutico. En todas las variables continuas se estudiará su posible distribución normal con el test K-S utilizando los test paramétricos cuando dicho test lo apruebe. Además, se utilizarán técnicas de regresión lineal, coeficiente de correlación de Pearson, para estimar relación entre variables cuantitativas y para estudiar la relación entre medias de variables cuantitativas el test t de Student, la U de Mann-Whitney o el análisis de varianza (ANOVA). Para el estudio de la relación entre variables cualitativas se utilizará el test de la χ2 y la prueba exacta de Fisher. Se realizará un análisis multivariante mediante regresión múltiple o logística según proceda para determinar el riesgo ajustado de presentar las variables de resultado. Para determinar la eficacia de la intervención se calculará el riesgo relativo con su intervalo de confianza del 95%. Para determinar la significatividad estadística se establecerá un nivel de 0.05. Los datos se analizarán con el programa SPSS 21.0.

Limitaciones

Una de las principales limitaciones en los ensayos clínicos aleatorizados hasta ahora es la valoración por parte de sujetos ciegos a la intervención, pues habitualmente los profesionales que atienden a los pacientes a la vez son los que realizan las mediciones de las variables. Además, al ser una población tan compleja y con importante deterioro funcional y cognitivo también se dificulta tanto la realización correcta como la monitorización de los resultados. Sin embargo, esta población habitualmente ha sido excluida de los ensayos clínicos por dichos motivos con lo cual es todavía más importante su inclusión en grupos de estudios para poder valorar la modificación de sus patrones asistenciales.

A pesar de que las condiciones en las que los pacientes son captados y la corta duración de la intervención favorecen la disponibilidad de la información de seguimiento, es necesario considerar la posible pérdida de individuos, inherente a los estudios longitudinales. El elevado número de individuos atendidos anualmente por las consultas de Geriatría y Medicina Interna de los dos centros participantes en el estudio deberían garantizar un tamaño muestral suficiente en este proyecto, a pesar de la limitación mencionada.

Otra limitación importante constituye el hecho que no se puede controlar de forma exacta que grado de seguimiento del programa que realizan los pacientes asignados al grupo intervención en el domicilio. Es conocido que los programas de ejercicio en domicilio plantean problemas en cuanto adherencia. En vistas a minimizar este problema se proporcionará material para que se registre el número de sesiones de entrenamiento semanales que realizan. Los pacientes que no puedan acudir a su hospital de referencia para las valoraciones al mes y a los 3 meses, se les hará un seguimiento telefónico coincidiendo con estas valoraciones y se les interrogará sobre la adherencia al entrenamiento y les pasará las escalas que se pueden hacer vía telefónica de cara a realizar un análisis ajustado a la realidad.
